# Clinical management of iron deficiency anemia in Japan: iron prescription patterns, treatment effectiveness, and assessments

**DOI:** 10.1007/s12185-024-03801-4

**Published:** 2024-05-28

**Authors:** Miyako Kosugi, Ryo Takezawa, Shun Shiota, Masaru Tsuchikawa, Katsuya Ikuta

**Affiliations:** 1 Data Science Department, Zeria Pharmaceutical Co., Ltd, 10-11 Nihonbashi Kobuna-Cho, Chuo-Ku, Tokyo, 103-8351 Japan; 2 Program Promotion Department, Hokkaido Blood Center, Sapporo, Japan

**Keywords:** Iron deficiency anemia, Iron replacement therapy, Ferric carboxymaltose, Real-world database, Japan

## Abstract

Iron deficiency anemia is one of the most common types of anemia, but real-world clinical management practices in Japan are unclear. This study retrospectively explored iron prescription patterns, treatment effectiveness, and assessments. Patients with at least one treatment period between September 2020 and September 2022 were included and classified into three groups (ferric carboxymaltose [FCM]: 7437 patients, saccharated ferric oxide [SFO]: 98,648 patients, and oral iron: 359,547 patients). Iron-related laboratory values over time and testing proportions were evaluated. Median baseline hemoglobin levels were lowest with FCM (FCM: 8.10 g/dL, SFO: 8.70 g/dL, oral iron: 9.70 g/dL), but changes in hemoglobin levels by 12 weeks were greatest with FCM (FCM: 3.20 g/dL, SFO: 2.60 g/dL, oral iron: 1.70 g/dL). The median serum ferritin level at 8 weeks after FCM treatment was 43.70 ng/mL for ≤500 mg, versus 123.30 ng/mL for >500 to ≤1500 mg. All groups had a low proportion of serum ferritin and transferrin saturation (TSAT) testing at diagnosis (<38%), which decreased further for post-treatment assessment (<24%). This study suggests the importance of prescribing an appropriate total iron cumulative dose per the package insert, along with diagnosis and assessments based on serum ferritin/TSAT.

**Supplementary Information:**

The online version contains supplementary material available at 10.1007/s12185-024-03801-4.

## Introduction

Iron deficiency anemia (IDA) is one of the most common types of anemia. It is a condition caused by the lack of iron required for the synthesis of hemoglobin (Hb) and occurs more frequently in females, due to regular blood loss during menstruation and significantly additional iron needs during adolescence and pregnancy [1, 2]. While the major cause of IDA in males and postmenopausal females is gastrointestinal bleeding [3], it has also been reported to be associated with post-gastrointestinal surgery, as well as a variety of diseases, including inflammatory bowel disease (IBD), cancer, heart failure (HF), and chronic kidney disease (CKD) [4, 5]. In recent years, treating IDA has also been suggested to reduce fatigue and the risk of psychiatric disorders, including depression [6, 7].

IDA can be treated with oral or intravenous (IV) iron preparations. Oral iron preparations are widely used in Japan as the first-line treatment because they are inexpensive and convenient. However, oral iron preparations cause gastrointestinal adverse effects in 10–20% of patients, and require at least 3 months to recover iron stores, often leading to poor adherence and unsatisfactory outcomes [8–10]. On the other hand, IV iron therapy is recommended when oral iron preparations cannot be tolerated due to gastrointestinal adverse effects or if there is a poor response to oral iron, such as in the presence of ongoing blood loss or gastrointestinal malabsorption [8]. IV iron preparations can replenish the iron stores rapidly, and the therapy can be completed in a shorter period than oral iron preparations [9, 10]. The only available IV iron preparation in Japan had been saccharated ferric oxide (SFO), until ferric carboxymaltose (FCM), which can be administered at higher doses, was newly available in Japan in September 2020. The total cumulative dose when using SFO or FCM is calculated based on Hb levels and body weight when the treatment started. According to these calculations, most patients require more than 1000 mg as the total cumulative dose, although the calculation method differs between SFO and FCM. The daily dose of SFO is 40–120 mg; therefore, frequent visits and vascular punctures are required to achieve the total cumulative dose of 1000 mg or more. On the other hand, the dose of FCM is 500 mg once a week, and FCM is given weekly until the patient’s calculated total cumulative dose (maximum: 1500 mg) is reached; thus, only 2–3 administrations are required to achieve the total cumulative dose of 1000 mg or more. However, the iron prescription pattern and whether adequate iron dose is prescribed in the actual clinical practice in Japan has not been clarified, and a survey of the current practice is warranted. In the U.S., the pattern of IV iron dosing, including FCM, has already been surveyed, showing that a higher total cumulative dose of iron was associated with improved Hb levels and reduced needs for further iron therapy [11]. However, because the types and dosages of available iron preparations in Japan differ from the U.S., a survey using Japanese data is needed.

For the diagnosis and post-treatment assessments of IDA, it is recommended to use serum ferritin and transferrin saturation (TSAT), indicators of iron deficiency, in addition to Hb [10, 12]. Serum iron, another indicator of iron deficiency, has low specificity of IDA because it has diurnal variation and decreases in anemia of chronic disease as well as IDA [8]. Serum ferritin reflects the total iron stores in the reticuloendothelial system, such as the liver. However, it can be elevated by other causes, such as inflammation, and should be interpreted cautiously [2, 13]. TSAT represents the amount of iron available for hematopoiesis, and a low TSAT indicates a shortage of iron supply to support normal erythropoiesis. It is expressed as the ratio of serum iron to total iron binding capacity (TIBC), which is complicated to calculate during routine clinical practice [8, 14]. A French study reported that serum ferritin and TSAT were rarely measured pre- and post-iron replacement therapy [15]. The current clinical practice of testing serum ferritin and TSAT in Japan is still unclear.

The objective of this study is to clarify iron prescription patterns, treatment effectiveness, and diagnostic and post-treatment assessments of IDA under current clinical practice in Japan using real-world data.

## Materials and methods

### Data source

This is a retrospective observational study using the database of Medical Data Vision Co., Ltd. (MDV; Tokyo, Japan). The MDV database collects inpatient and outpatient insurance claims, blood tests, and inpatient discharge data from acute care hospitals adopting the Japanese Diagnosis Procedure Combination/Per-Diem Payment System (DPC/PDPS). As of September 2022, this database covers 41.2 million patients and 472 facilities (about 27% of acute care hospitals in Japan), making it one of the largest commercial databases in Japan [16], and was used for many studies, including surveys exploring treatment patterns and evaluating risk factors in various diseases [17–19].

### Study design (Fig. 1)

The inclusion criteria for this study were patients who received at least one iron prescription between September 1, 2020, and September 30, 2022, and patients who could constitute a Treatment Period. A Treatment Period refers to a series of treatments with the same iron group, defined as the time period of continuous prescriptions of iron classified in the same group without an interval of 42 days or more between prescriptions. The eligible patients were classified into the FCM, SFO, and oral iron groups according to the iron prescribed in the Treatment Period. For the IV iron group, this study only included FCM and SFO, because they were the only commercially available IV iron preparations during the evaluated data period. For example, if there were no iron prescriptions within 42 days both before and after a single FCM prescription, the date of this prescription is defined as the Treatment Period in the FCM group. In this case, the start date of Treatment Period (STP) and the end date of Treatment Period (ETP) would be the same day, and the duration of the Treatment Period would be 1 day. Since oral iron preparations are poorly absorbed when combined with IV iron preparations [8], the IV iron group (FCM and SFO group) was allowed to include oral iron prescriptions within the Treatment Period. This analysis used only the first Treatment Period during the evaluated data period, and the Index date was defined as the start date of the first Treatment Period. Exclusion criteria in this study were as follows: (1) Patients with an iron prescription within 42 days before the Index date, due to target patients who received new iron prescriptions. However, in the case of the IV iron group, patients prescribed oral iron within 42 days before the Index date were not excluded. (2) Patients on dialysis within 180 days before the Index date, because the target Hb levels in dialysis patients differ from the Hb normal range in non-dialysis patients [20].

**Fig. 1:**
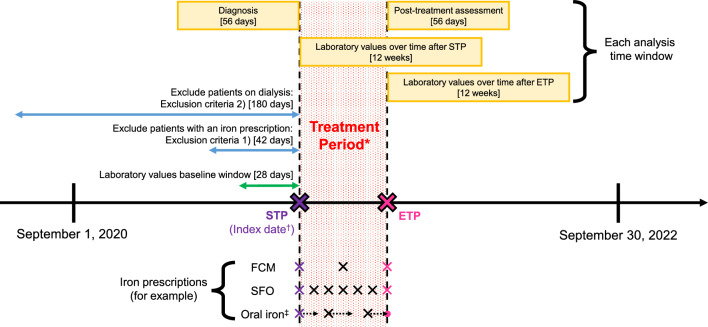
Study design. * Treatment Period refers to a series of treatments with the same iron group, defined as the time period of continuous prescriptions of iron classified in the same group without an interval of 42 days or more between prescriptions. ^†^ Index date was defined as the start date of the first Treatment Period. ^‡^ The prescriptions of oral iron group were defined as the date of prescription + the number of days of prescription (shown by dotted arrows). ETP, The end date of Treatment Period; FCM, ferric carboxymaltose; SFO, saccharated ferric oxide; STP, The start date of Treatment Period

Drug prescriptions, disease names, and medical practices used in this study were defined using the International Statistical Classification of Diseases and Related Health Problems 10th Revision (ICD-10), European Pharmaceutical Market Research Association (EphMRA) Anatomical Therapeutic Chemical (ATC) classification, and Japan-specific standardized codes (Tables S1–S3). For the inpatient-outpatient classification, patients with all medical practices received in an inpatient setting during the Treatment Period were classified as inpatients. Patients who did not meet the definition of inpatients were classified as outpatients.

### Outcome measures

The effectiveness of iron replacement therapy was assessed by laboratory values (Hb, serum ferritin, and TSAT) and change from baseline of these values. The FCM group was categorized according to their cumulative prescription doses during the Treatment Period as ≤500 mg, or >500 and ≤1500 mg. Assessment time points were as follows: (1) baseline (equal to STP), (2) ETP, and (3) 2, 4, 8, and 12 weeks from STP or ETP. The analyzed population was the overall eligible population and the subgroups included inpatient and outpatient.

Diagnosis and post-treatment assessments of IDA were evaluated by the proportion of tests performed (Hb, serum iron, serum ferritin, and TSAT) within 56 days before STP and within 56 days after ETP. The analyzed population included the overall eligible population and patients from specific disease area: gynecology, obstetrics, IBD, gastrointestinal bleeding or malabsorption (other than IBD), cancer, HF, and non-dialysis-dependent CKD (NDD-CKD).

### Statistical analysis

Summary statistics are presented as frequency and proportion (%) for categorical variables, and median and interquartile range (IQR) for continuous variables. Missing data was not imputed. The baseline for laboratory values was defined as terms within 28 days before the Index date. The windows for each assessment time point for laboratory values over time were as follows: ETP—6 days after ETP; 2 weeks—7–20 days after STP or ETP; 4 weeks—21–41 days after STP or ETP; 8 weeks—42–69 days after STP or ETP; 12 weeks—70–98 days after STP or ETP. If multiple laboratory values were assessed in the time window for a particular assessment time point, the values closest to each assessment time point were used. In addition, as a sensitivity analysis, we excluded patients who had any oral iron prescriptions in the IV iron groups, and those who had blood transfusions or autologous blood donation in all groups from the overall eligible population within 42 days before the Index date, in order to evaluate the impact of each medical treatment on the results of the main analysis. All statistical analyses were performed using SAS^®^ ver. 9.4 (SAS Institute Inc., Cary, NC, USA).

### Ethical statement

This study was conducted in accordance with the Ethical Guidelines for Medical and Biological Research Involving Human Subjects and the Declaration of Helsinki for applicable items, and was approved by the Ethical Committee of MINS, a non-profit organization (approval number, 230212; approval date, May 18, 2023). Informed consent was not required because all data were anonymized.

## Results

### Study population

**Fig. 2:**
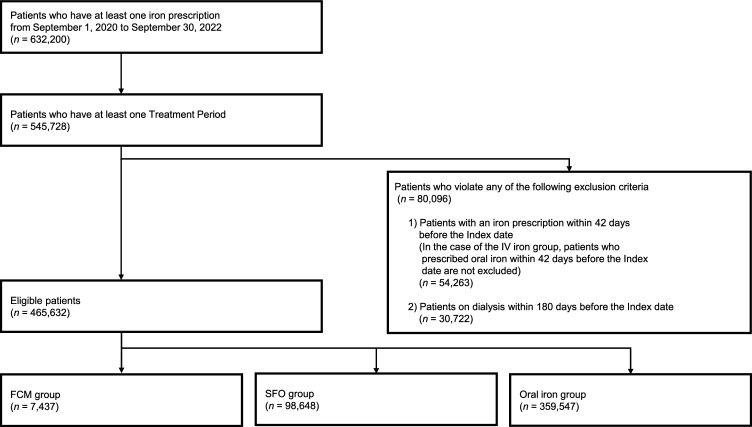
Patient flowchart in this study. FCM, ferric carboxymaltose; SFO, saccharated ferric oxide

The patient flowchart is shown in Fig. 2. A total of 632,200 patients received at least one iron prescription from September 1, 2020, to September 30, 2022. Of these, the number of eligible patients in each group who met the inclusion criteria and did not violate any of the exclusion criteria was as follows: 7437 in the FCM group, 98,648 in the SFO group, and 359,547 in the oral iron group.

### Patient characteristics

The demographic and baseline patient characteristics of interest are summarized in Table 1. There were more female patients than male patients in all groups, with 76.3% in the FCM group, 61.8% in the SFO group, and 70.1% in the oral iron group. The median age (IQR) was 50.0 (40.0–73.0) years for the FCM group, 71.0 (46.0–82.0) years for the SFO group, and 65.0 (38.0–81.0) years for the oral iron group. The FCM group included more young to middle-aged patients (aged 20–54) compared to the other groups: 57.4% in the FCM group, 32.0% in the SFO group, and 39.6% in the oral iron group. Most patients were classified as weighing 35 kg or more: 97.6% in the FCM group, 96.6% in the SFO group, and 92.2% in the oral iron group. The proportion of patients who had received a blood transfusion within 42 days before the Index date was 11.3% in the FCM group, 18.4% in the SFO group, and 6.7% in the oral iron group. The proportion of patients who had received blood transfusions in the Treatment Period was 6.6% in the FCM group, 21.2% in the SFO group, and 7.7% in the oral iron group. In the IV iron group, the proportion of patients who also received an oral iron preparation within 42 days before the Index date to ETP was 27.6% in the FCM group and 32.6% in the SFO group. The proportion of patients with baseline Hb levels <8 g/dL was 46.5% in the FCM group, 33.0% in the SFO group, and 14.3% in the oral iron group, while the proportion of patients with baseline Hb levels ≥8 g/dL was 53.5% in the FCM group, 67.0% in the SFO group, and 85.7% in the oral iron group. The proportion of patients classified as outpatients was 61.9% in the FCM group, 16.8% in the SFO group, and 65.6% in the oral iron group.

**Table 1:** Patient characteristics

	FCM	SFO	Oral iron
	(*N* = 7437)	(*N* = 98,648)	(*N* = 359,547)
Female gender, *n* (%)	5676 (76.3)	60,986 (61.8)	252,162 (70.1)
*Age (years)*			
Median (IQR)	50.0 (40.0–73.0)	71.0 (46.0–82.0)	65.0 (38.0–81.0)
<20 years, *n* (%)	105 (1.4)	1109 (1.1)	16,990 (4.7)
≥20 and <45 years, *n* (%)	2543 (34.2)	22,091 (22.4)	101,939 (28.4)
≥45 and <55 years, *n* (%)	1728 (23.2)	9517 (9.6)	40,448 (11.2)
≥55 and <65 years, *n* (%)	490 (6.6)	7843 (8.0)	19,638 (5.5)
≥65 years, *n* (%)	2571 (34.6)	58,088 (58.9)	180,532 (50.2)
*Weight (kg)*			
Median (IQR)	55.10 (47.90–64.00)	54.90 (46.80–63.60)	53.00 (44.30–62.00)
*n* (%)	3068 (41.3)	78,152 (79.2)	164,180 (45.7)
<25 kg	3 (0.1)	175 (0.2)	6907 (4.2)
≥25 and <35 kg	72 (2.3)	2459 (3.1)	5853 (3.6)
≥35 and <70 kg	2575 (83.9)	64,865 (83.0)	132,637 (80.8)
≥70 kg	418 (13.6)	10,653 (13.6)	18,783 (11.4)
*Blood transfusion within 42 days before the Index date*			
*n* (%)	840 (11.3)	18,166 (18.4)	24,126 (6.7)
>400 mL	572 (68.1)	12,389 (68.2)	15,028 (62.3)
*Blood transfusion in the Treatment Period*			
*n* (%)	490 (6.6)	20,903 (21.2)	27,850 (7.7)
>400 mL	270 (55.1)	13,195 (63.1)	16,195 (58.2)
*Autologous blood donation in the Treatment Period*			
*n* (%)	286 (3.8)	4126 (4.2)	10,946 (3.0)
>400 mL	40 (14.0)	1901 (46.1)	4549 (41.6)
*Hb (g/dL)*			
Median (IQR)	8.10 (7.10–9.10)	8.70 (7.60–10.00)	9.70 (8.60–10.80)
*n* (%)	876 (11.8)	11,034 (11.2)	38,692 (10.8)
<8 g/dL	407 (46.5)	3640 (33.0)	5543 (14.3)
≥8 g/dL	469 (53.5)	7394 (67.0)	33,149 (85.7)
<10 g/dL	778 (88.8)	8262 (74.9)	22,496 (58.1)
≥10 g/dL	98 (11.2)	2772 (25.1)	16,196 (41.9)
*Serum ferritin (ng/mL)*			
Median (IQR)	17.80 (7.00–62.40)	29.50 (11.00–94.00)	21.00 (8.10–76.00)
*n* (%)	267 (3.6)	2810 (2.8)	9344 (2.6)
<12 ng/mL	104 (39.0)	741 (26.4)	3253 (34.8)
≥12 ng/mL	163 (61.0)	2069 (73.6)	6091 (65.2)
*Serum iron (µg/dL)*			
Median (IQR)	20.00 (11.00–39.00)	24.00 (13.00–46.00)	27.00 (17.00–48.00)
*n* (%)	391 (5.3)	4848 (4.9)	14,705 (4.1)
*TSAT (%)*			
Median (IQR)	6.40 (3.28–14.84)	8.62 (4.24–19.32)	8.19 (4.46–16.54)
*n* (%)	307 (4.1)	2984 (3.0)	9332 (2.6)
<20%	248 (80.8)	2262 (75.8)	7516 (80.5)
≥20%	59 (19.2)	722 (24.2)	1816 (19.5)
*CRP (mg/dL)*			
*n* (%)	694 (9.3)	9574 (9.7)	26,979 (7.5)
≤1.0 mg/dL	419 (60.4)	5007 (52.3)	16,394 (60.8)
>1.0 mg/dL	275 (39.6)	4567 (47.7)	10,585 (39.2)
*Inpatient or outpatient, n (%)*			
Inpatient	2835 (38.1)	82,044 (83.2)	123,818 (34.4)
Outpatient	4602 (61.9)	16,604 (16.8)	235,729 (65.6)
*Comorbidities*^*a*^*, n (%)*			
Gynecology	2076 (27.9)	9934 (10.1)	34,329 (9.5)
Heavy menstrual bleeding^b^	692 (33.3)	2762 (27.8)	8599 (25.0)
Abnormal uterine bleeding^b^	308 (14.8)	1796 (18.1)	4829 (14.1)
Fibroids^b^	1473 (71.0)	6950 (70.0)	24,708 (72.0)
Adenomyosis or endometriosis^b^	623 (30.0)	2600 (26.2)	8064 (23.5)
Uterine fibroid-related surgery^b^	185 (8.9)	2748 (27.7)	2523 (7.3)
Obstetrics	858 (11.5)	11,929 (12.1)	35,069 (9.8)
Pregnancy^b^	277 (32.3)	3770 (31.6)	18,394 (52.5)
Childbirth and the puerperium^b^	743 (86.6)	10,756 (90.2)	20,144 (57.4)
IBD	607 (8.2)	2077 (2.1)	4249 (1.2)
UC^b^	319 (52.6)	1433 (69.0)	3163 (74.4)
CD^b^	319 (52.6)	706 (34.0)	1214 (28.6)
Gastrointestinal bleeding (excluding IBD)	852 (11.5)	22,862 (23.2)	20,143 (5.6)
Gastrointestinal malabsorption (excluding IBD)	75 (1.0)	1481 (1.5)	1491 (0.4)
Heart failure	803 (10.8)	18,044 (18.3)	60,666 (16.9)
Cancer	1503 (20.2)	26,258 (26.6)	64,805 (18.0)
NDD-CKD	381 (5.1)	10,867 (11.0)	37,994 (10.6)
*Total iron dose in the Treatment Period (mg)*			
Median (IQR)	500.00 (500.00–1000.00)	280.00 (160.00–480.00)	2800.00 (1400.00–6000.00)
*Total IV iron dose in the Treatment Period by prescription dose (mg)*			
≤500 mg, *n* (%)	4564 (61.4)	74,573 (75.6)	–
>500 and ≤1000 mg, *n* (%)	1540 (20.7)	17,482 (17.7)	–
>1000 and ≤1500 mg, *n* (%)	1220 (16.4)	3986 (4.0)	–
>1500 mg, *n* (%)	113 (1.5)	2607 (2.6)	–
*Duration in the Treatment Period (days)*			
Median (IQR)	1.0 (1.0–10.0)	5.0 (2.0–8.0)	30.0 (14.0–67.0)
*Mean iron dose per a day in the Treatment Period (mg)*			
Median (IQR)	500.00 (107.14–500.00)	80.00 (40.00–80.00)	100.00 (67.39–105.00)
*Combined with oral iron in IV iron groups from 42 days before the Index date to ETP*			
*n* (%)	2052 (27.6)	32,180 (32.6)	–

*CD* Crohn’s disease, *CRP* C-reactive protein, *ETP* The end date of Treatment Period, *FCM* ferric carboxymaltose, *Hb* hemoglobin, *IBD* inflammatory bowel disease, *IQR* interquartile range, *IV* intravenous, *NDD-CKD* non-dialysis dependent chronic kidney disease, *SFO* saccharated ferric oxide, *TSAT* transferrin saturation, *UC* ulcerative colitis

^a^Multiple choice allowed

^b^The denominator of *n* (%) is the patients who applied the superordinate disease area in each iron group

### Prescribed iron dose and duration

For the IV iron group, the median (IQR) total prescribed iron dose in the Treatment Period was 500.00 (500.00–1000.00) mg for the FCM group and 280.00 (160.00–480.00) mg for the SFO group. For the oral iron group, the median (IQR) mean daily prescribed iron dose in the Treatment Period was 100.00 (67.39–105.00) mg. The median (IQR) duration in the Treatment Period for each group was 1.0 (1.0–10.0) days for the FCM group, 5.0 (2.0–8.0) days for the SFO group, and 30.0 (14.0–67.0) days for the oral iron group (Table 1).

### Iron prescriptions by disease area

The proportion of patients prescribed oral iron among the overall eligible population in each disease area was 77.2% in NDD-CKD, 76.3% in HF, and 74.1% in gynecology; The proportion of patients prescribed IV iron was 53.8% in gastrointestinal bleeding or malabsorption (other than IBD), and 38.7% in IBD. The proportion of patients prescribed FCM among the IV iron group was 22.6% in IBD, 17.3% in gynecology, and 6.7% in obstetrics (Table S4).

### Outcome measures

#### Effectiveness of iron replacement therapy

**Fig. 3:**
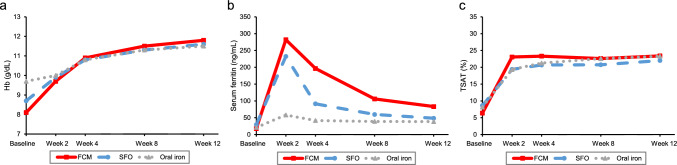
Laboratory values over time after STP (median). **a** Hb, **b** serum ferritin, **c** TSAT. FCM, ferric carboxymaltose; Hb, hemoglobin; SFO, saccharated ferric oxide; STP, The start date of Treatment Period; TSAT, transferrin saturation

Figure 3 shows the laboratory values over time after STP for each group. The median (IQR) baseline Hb levels were 8.10 (7.10–9.10) g/dL in the FCM group, 8.70 (7.60–10.00) g/dL in the SFO group, and 9.70 (8.60–10.80) g/dL in the oral iron group, with the FCM group having the lowest baseline Hb level. The median change in Hb levels from baseline to 12 weeks was 3.20 g/dL in the FCM group, 2.60 g/dL in the SFO group, and 1.70 g/dL in the oral iron group. The median serum ferritin (IQR) for each group at baseline, 2 weeks, and 12 weeks was 17.80 (7.00–62.40) ng/mL, 282.00 (188.60–495.00) ng/mL, and 83.20 (30.30–217.20) ng/mL in the FCM group, 29.50 (11.00–94.00) ng/mL, 232.40 (91.90–501.00) ng/mL, and 48.60 (20.65–148.00) ng/mL in the SFO group, and 21.10 (8.10–76.00) ng/mL, 58.20 (27.96–154.10) ng/mL, and 38.53 (20.00–78.00) ng/mL in the oral iron group, respectively. The median (IQR) TSAT for each group at baseline, 2 weeks, and 12 weeks was 6.40 (3.28–14.84)%, 23.06 (15.28–33.04)% and 23.39 (13.04–30.45)% in the FCM group, 8.62 (4.24–19.32)%, 19.39 (12.35–30.09)%, and 22.00 (12.50–31.58)% in the SFO group, and 8.19 (4.46–16.54)%, 19.33 (11.26–31.67)%, and 23.28 (14.84–33.22)% in the oral iron group, respectively (Table S5).

**Fig. 4:**
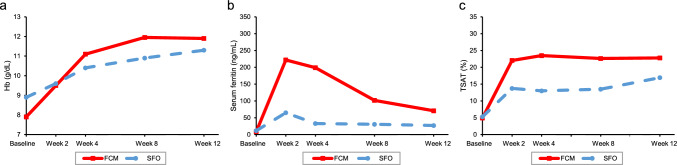
Laboratory values over time after STP for outpatients (median). **a** Hb, **b** serum ferritin, **c** TSAT. FCM, ferric carboxymaltose; Hb, hemoglobin; SFO, saccharated ferric oxide; STP, The start date of Treatment Period; TSAT, transferrin saturation

Figure 4 shows the laboratory values over time after STP for outpatients in the IV iron group. The median (IQR) Hb levels at baseline, 2 weeks, and 12 weeks was 7.90 (6.90–9.20) g/dL, 9.50 (8.50–10.50) g/dL, and 11.90 (10.30–13.00) g/dL in the FCM group and 8.90 (7.40–10.60) g/dL, 9.60 (8.40–11.00) g/dL, and 11.30 (9.70–12.60) g/dL in the SFO group, respectively. The median (IQR) serum ferritin at baseline, 2 weeks, and 12 weeks was 8.90 (4.30–22.50) ng/mL, 222.00 (159.10–375.74) ng/mL, and 70.80 (30.00–212.00) ng/mL in the FCM group and 11.70 (5.00–29.20) ng/mL, 65.00 (26.20–150.00), and 27.00 (13.90–59.00) ng/mL in the SFO group, respectively. The median (IQR) TSAT at baseline, 2 weeks, and 12 weeks was 4.95 (2.68–11.89)%, 22.03 (14.36–31.41)%, and 22.79 (12.73–29.20)% in the FCM group and 5.29 (2.72–13.79)%, 13.73 (6.20–29.67)%, and 16.92 (9.40–28.82)% in the SFO group, respectively. The results of the prescribed iron dose and duration by outpatients are as follows: The median (IQR) total prescribed iron dose in the Treatment Period and the median duration of the Treatment Period was 500.00 (500.00–1000.00) mg and 1.0 (1.0–15.0) days for the FCM group, and 120.00 (80.00–240.00) mg and 3.0 (1.0–20.0) days for the SFO group, respectively. The proportion of patients who also received an oral iron preparation in addition to the IV iron preparation from 42 days before the Index date to ETP was 29.2% in the FCM group and 53.3% in the SFO group (Table S6).

In the sensitivity analysis, the number of patients in each group is as follows: 4358 in the FCM group, 41,061 in the SFO group, and 305,318 in the oral iron group. The median (IQR) baseline Hb levels were 8.20 (7.30–9.30) g/dL in the FCM group, 9.30 (8.10–10.50) g/dL in the SFO group, and 9.80 (8.80–10.80) g/dL in the oral iron group, and the median change in Hb levels from baseline to 12 weeks after STP was 3.20 g/dL in the FCM group, 1.90 g/dL in the SFO group, and 1.80 g/dL in the oral iron group (data not shown).

#### Effectiveness of FCM by prescription dose

Table 2 shows the laboratory values and the change from baseline to 12 weeks after ETP by prescription dose for the FCM group. The proportion of patients with a total prescribed iron dose of ≤500 mg, and >500 and ≤1500 mg in the Treatment Period was 61.4% and 37.1%, respectively (Table 1). For the patients with a total prescribed iron doses ≤500 mg, the median (IQR) Hb levels at baseline and 12 weeks after ETP were 8.30 (7.40–9.20) g/dL and 11.50 (10.00–12.70) g/dL, respectively. For the patients with a total prescribed iron doses >500 and ≤1500 mg, the median (IQR) Hb values at baseline and 12 weeks after ETP were 7.60 (6.50–8.70) g/dL and 12.20 (11.00–13.60) g/dL, respectively. For the patients with a total prescribed iron doses ≤500 mg, the median (IQR) serum ferritin at baseline and 8 weeks after ETP was 24.35 (9.20–79.95) ng/mL and 43.70 (32.00–106.00) ng/mL, respectively. For the patients with a total prescribed iron doses >500 and ≤1500 mg, the median (IQR) serum ferritin at baseline and 8 weeks after ETP were 10.40 (5.20–37.00) ng/mL and 123.30 (64.00–242.50) ng/mL, respectively.

**Table 2:** Laboratory values and changes by prescription dose for the FCM group

	≤500 mg				>500 and ≤1500 mg			
	Laboratory values		Changes^a^		Laboratory values		Changes^a^	
	*n*	Median (IQR)	*n*	Median (IQR)	*n*	Median (IQR)	*n*	Median (IQR)
*Hb*								
Baseline	566	8.30 (7.40–9.20)	–	–	296	7.60 (6.50–8.70)	–	–
ETP^b^	–	–	–	–	211	9.20 (8.10–9.90)	210	1.30 (0.20–2.70)
After ETP								
Week 2	288	10.00 (9.10–10.90)	283	1.80 (0.60–2.90)	162	10.50 (9.60–11.50)	159	2.90 (1.30–4.20)
Week 4	274	11.00 (10.10–12.10)	268	2.50 (1.10–3.90)	146	11.50 (10.10–12.50)	145	3.70 (1.90–5.00)
Week 8	184	11.10 (10.10–12.60)	179	2.50 (1.20–4.20)	112	11.85 (10.20–13.10)	112	3.80 (2.05–5.45)
Week 12	147	11.50 (10.00–12.70)	142	2.70 (1.40–4.20)	78	12.20 (11.00–13.60)	77	4.00 (2.50–5.80)
*Serum ferritin*								
Baseline	160	24.35 (9.20–79.95)	–	–	101	10.40 (5.20–37.00)	–	–
ETP^b^	–	–	–	–	37	279.50 (171.00–496.30)	25	251.80 (144.60–462.90)
After ETP								
Week 2	29	273.00 (198.50–428.20)	16	240.65 (193.35–449.00)	38	290.10 (197.70–452.04)	21	291.80 (216.00–469.30)
Week 4	52	135.50 (44.00–261.00)	37	100.40 (26.00–181.00)	21	196.80 (71.00–320.00)	18	151.85 (43.70–375.00)
Week 8	25	43.70 (32.00–106.00)	15	20.00 (4.00–42.80)	20	123.30 (64.00–242.50)	12	83.85 (58.45–236.50)
Week 12	24	52.95 (23.36–154.50)	15	37.00 (2.00–126.00)	14	45.50 (30.00–212.00)	9	32.80 (7.80–168.00)
*TSAT*								
Baseline	191	7.27 (4.25–15.63)	–	–	110	5.74 (2.73–14.12)	–	–
ETP^b^	–	–	–	–	40	21.90 (14.11–30.94)	26	15.85 (7.67–29.36)
After ETP								
Week 2	42	23.29 (16.90–32.57)	29	16.27 (11.05–23.85)	26	29.63 (18.36–40.11)	20	24.77 (13.35–29.97)
Week 4	56	23.08 (14.76–31.66)	38	11.91 (1.43–21.92)	26	21.58 (17.77–32.37)	21	15.92 (3.29–20.48)
Week 8	26	23.30 (12.80–30.41)	19	13.30 (6.94–21.20)	19	23.08 (16.21–30.20)	14	12.88 (0.39–21.68)
Week 12	28	23.15 (10.41–30.01)	21	15.38 (2.82–21.44)	14	22.70 (18.87–34.55)	9	18.24 (10.93–32.27)

*ETP* The end date of Treatment Period, *FCM* ferric carboxymaltose, *Hb* hemoglobin, *IQR* interquartile range, *TSAT* transferrin saturation

^a^Patients who have both the value of baseline and the value of each assessment time point are evaluated for “Changes”

^b^No data at ETP in ≤500 mg dose group because baseline and ETP are considered as the same assessment point

#### Diagnosis and post-treatment assessment of IDA

**Fig. 5:**
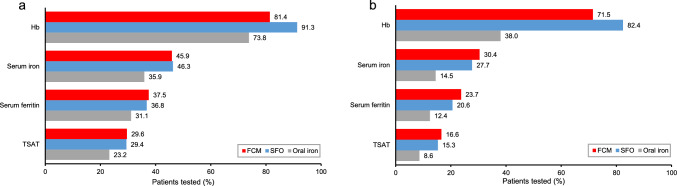
Proportion of patients tested for diagnostic and post-treatment assessments of IDA. **a** Diagnosis, **b** post-treatment assessment.: FCM, ferric carboxymaltose; Hb, hemoglobin; IDA, iron deficiency anemia; SFO, saccharated ferric oxide; TSAT, transferrin saturation

Figure 5 shows the proportions of patients tested for diagnostic and post-treatment assessments of IDA. The proportion of Hb testing was high in the IV iron group for both diagnostic and post-treatment assessments: 81.4% and 71.5% in the FCM group, and 91.3% and 82.4% in the SFO group, respectively. The oral iron group had a lower Hb testing proportion for post-treatment assessment than for diagnosis: 73.8% for diagnosis and 38.0% for post-treatment assessment. The serum iron testing in all groups has the second highest proportion after Hb testing: for diagnostic and post-treatment assessments, 45.9% and 30.4% in the FCM group, 46.3% and 27.7% in the SFO group, and 35.9% and 14.5% in the oral iron group, respectively. The proportion of serum ferritin testing was lower than that of serum iron testing in all groups: for diagnostic and post-treatment assessments, 37.5% and 23.7% in the FCM group, 36.8% and 20.6% in the SFO group, and 31.1% and 12.4% in the oral iron group, respectively. The proportion of TSAT testing was slightly lower than that of serum ferritin testing in all groups: for diagnostic and post-treatment assessments, 29.6% and 16.6% in the FCM group, 29.4% and 15.3% in the SFO group, and 23.2% and 8.6% in the oral iron group, respectively.

The disease areas with the highest test proportion of serum ferritin at diagnosis were NDD-CKD in the FCM and oral iron groups (65.1% and 51.0%, respectively), and HF in the SFO group (55.6%). Each group had the lowest test proportion of serum ferritin in obstetrics (FCM group: 3.4%, SFO group: 2.6%, oral iron group: 1.1%), followed by gynecology (FCM group: 26.1%, SFO group: 16.4%, oral iron group: 16.6%). The disease areas with the highest test proportion of TSAT at diagnosis were NDD-CKD in the FCM and oral iron groups (56.4% and 40.3%, respectively), and HF in the SFO group (43.7%). Each group had the lowest test proportion of TSAT in obstetrics (FCM group: 2.9%, SFO group: 2.1%, oral iron group: 0.7%), followed by gynecology (FCM group: 21.3%, SFO group: 14.6%, oral iron group: 12.7%) (Table S7).

## Discussion

First-line oral iron preparations were more commonly prescribed than IV iron preparations. In the IV iron group, SFO was prescribed more often than FCM, partly because the data period of this study covered approximately 2 years immediately after FCM was available, whereas SFO had already been on the market for half a century. Similar to previous reports [1, 2], the proportion of females was higher in all groups. In the IV iron group, both the proportion of blood transfusions performed and the proportion of combination therapy with oral iron were higher in the SFO group than in the FCM group, suggesting that IDA was more frequently treated with iron preparations alone in the FCM group. Among all groups, the FCM group had the highest proportion of patients with baseline Hb levels <8 g/dL, accounting for approximately 47% of the FCM group, and the oral iron group had the highest proportion of patients with baseline Hb levels ≥8 g/dL, accounting for approximately 86% of the oral iron group (Table 1). Oral iron preparations tended to be selected for the patients with higher Hb levels, while SFO was prescribed for those with lower Hb levels, and FCM was prescribed for those with even lower Hb levels. However, there were still 14% of patients in the oral iron group with baseline Hb levels <8 g/dL. It is also important to note that in principle, the Japanese insurance claims system requires FCM to be prescribed only to patients with Hb levels <8 g/dL, and if the patient has an Hb level ≥8 g/dL, justification for early high-dose iron supplementation is required [21]. Even then, FCM was prescribed to approximately 54% of patients with baseline Hb levels ≥8 g/dL (Table 1). This result showed that in real-world settings, healthcare professionals prescribed FCM to patients with Hb levels ≥8 g/dL after taking their conditions and/or comorbidities into considerations.

In terms of iron prescriptions in each disease area, the proportion of oral iron prescriptions was higher than that of IV iron prescriptions in most disease areas, except for gastrointestinal diseases. This is potentially because oral iron preparations are inappropriate for the compromised gastrointestinal tract, and therefore, IV iron preparations may be preferred for patients with these diseases. FCM was also prescribed in obstetrics, gynecology, and IBD. The majority of the FCM group patients in this study were aged 20–54, which is also the common age range for diseases in obstetrics, gynecology, and IBD, suggesting that higher doses of FCM may be preferred for conditions with chronic bleeding such as heavy menstrual bleeding and IBD (Table S8).

Regarding the laboratory values over time from STP for each group, the FCM group had the highest changes by 12 weeks with the lowest baseline Hb levels, suggesting that it improves Hb levels even for severe IDA patients. In the sensitivity analysis, similar results were obtained for the FCM group and the oral iron group, and there were almost no effects of blood transfusions, autologous blood donation, or oral iron prescription for the IV iron group. On the other hand, in the SFO group, the proportion of patients who received blood transfusions during the term was higher than in the other groups (21.2%). As a result of the sensitivity analysis, the change in Hb levels by 12 weeks for the SFO group was small (1.90 g/dL), suggesting that SFO prescribed alone may lead to an unsatisfactory response. Regarding serum ferritin levels, similar to previous reports [8], the IV iron group showed temporarily high levels (median serum ferritin at 2 weeks was 282.00 ng/mL in the FCM group and 232.40 ng/mL in the SFO group), but then slowly declined. Serum ferritin levels at 12 weeks were higher in the FCM group than in the SFO group, suggesting that the iron store was more adequately supplemented by FCM (Fig. 3; Table S5). TSAT improved to approximately 20% at 2 weeks in all groups and remained at the same level throughout the 12 weeks, suggesting that the iron available for hematopoiesis in the blood could be maintained by all three iron preparations (Fig. 3).

Most patients in the SFO group were classified as inpatients, whereas the FCM group had more patients classified as outpatients (Table 1). The high proportion of inpatients in the SFO group may be attributed to the need for frequent visits to achieve the total cumulative dose of 1000 mg or more, considering that the maximum daily dose of SFO is 120 mg. Regarding the laboratory value changes over time from STP for outpatients in the IV iron group, Hb levels in the FCM group were lower than the SFO group at baseline and comparable to the SFO group at 12 weeks. On the other hand, serum ferritin and TSAT at 2, 4, 8, and 12 weeks tended to be lower in the SFO group than FCM group (Fig. 4). The reason for this was presumably due to the difference in total cumulative doses; the median total prescription dose and median duration in the Treatment Period were 500 mg and 1 day in the FCM group, and 120 mg and 3 days in the SFO group, respectively (Table S6). SFO can be administered frequently in the inpatient setting, however, it may be difficult to frequently administer SFO in the outpatient setting, leading to inadequate iron levels in the patient. In contrast, FCM can provide 500 mg of iron supplementation once a week with only 2–3 visits to achieve the required iron level. FCM is convenient for both in- and outpatients, especially for patients who are unable to visit the hospital frequently, or when iron preparations need to be provided on an outpatient basis.

Regarding the total cumulative iron dose, 1000 or 1500 mg is required for patients in the FCM group whose weight is 35 kg or more at the start of the therapy. However, in real-world settings, approximately 60% of patients were prescribed a single 500 mg dose (Table 1). This means that the optimal dose of the FCM package insert was not given to the patients. Regarding the laboratory values over time from ETP by prescription doses in the FCM group, doses greater than 500 mg in the Treatment Period were prescribed for patients with low baseline Hb levels. The median serum ferritin levels were temporarily increased at 2 weeks. Subsequently, the levels at 8 weeks after ETP were 43.70 ng/mL for the ≤500 mg dose group and 123.30 ng/mL for the >500 and ≤1500 mg dose group. Although a single dose of FCM can significantly increase Hb levels, simply confirming increases in Hb levels is not enough to decide when to complete iron supplementation. The appropriate dosage described in the FCM package insert is determined based on the replenishment of iron stores in the body. Therefore, for longer-term iron stores in the patient, it is suggested that the proper dose of 1000 mg or 1500 mg be prescribed instead of a single 500 mg prescription (Table 2). In addition, the total cumulative dose of FCM does not require a complicated calculation like SFO and is determined by a combination of baseline Hb levels and body weight. In the future, we will need to disseminate the ways to use FCM appropriately.

All groups had a higher proportion of Hb testing, followed by serum iron testing. On the other hand, the proportion of serum ferritin and TSAT testing were lower. The results suggest that the measurement of serum ferritin and TSAT may not be prevalent for either the diagnosis or post-treatment assessments of IDA, and that serum iron may be used instead of serum ferritin and/or TSAT. To assess iron deficiency more accurately, measurements should include not only Hb and serum iron but also serum ferritin and TSAT. In cases of increased iron demand, iron deficiency due to chronic diseases or bleeding, and severe iron deficiency, IV iron preparations, which provide high and rapid iron repletion, should be considered as a treatment option. The proportions of all tests in the post-treatment assessments were lower than in the diagnosis, suggesting that the tests in the post-treatment assessments may not be considered important. It is noted that serum ferritin levels may not return to normal even if Hb levels improve and subjective symptoms of anemia disappear with the administration of iron supplements. In the case of IV iron preparations, serum ferritin levels were temporarily elevated immediately after the administration of IV iron, therefore reassessment 4 weeks after the treatment rather than immediately after administration is recommended [10, 22]. In the post-treatment assessments, the proportions of tests, especially Hb, were considerably lower in the oral iron group compared to the IV iron group. One of the possible causes was thought to be the definition of the analysis time window. ETP for the oral iron group was defined by the date of the last prescription + the number of days for the prescription, and therefore, patients did not always revisit the hospital at ETP (Fig. 5).

The proportion of serum ferritin and TSAT testing in each disease area was lower in gynecology and obstetrics and higher in HF and NDD-CKD. In gynecology and obstetrics, it was suggested that iron supplementation may be initiated without testing if iron deficiency is evident through bleeding. HF and CKD are known to be complicated by anemias other than iron deficiency [20, 23]; therefore, it is necessary to differentiate between IDA and non-IDA, making diagnostic methods using serum ferritin and TSAT more prevalent in HF and CKD than in other disease areas (Table S7).

This study has several limitations. First, this is an observational study using real-world data, and unmeasured data cannot be ascertained. Second, MDV data does not capture medical information in other hospitals. In addition, blood test data were provided only from a few hospitals, which may have biased the data; Hb, serum ferritin, and TSAT data were obtained from only 10.9%, 2.7%, and 2.7% of the overall eligible population, respectively. Finally, patients diagnosed with iron deficiency anemia without iron prescriptions were not included in this study, because we identified the eligible patients based on iron prescriptions.

## Conclusion

We clarified current clinical practice in Japan with regard to iron prescription patterns, treatment effectiveness, and diagnostic and post-treatment assessments of IDA using real-world database. The FCM group had the highest changes by 12 weeks with the lowest baseline Hb levels. There was still a proportion of patients, even those with Hb <8 g/dL, who were prescribed oral iron, where perhaps IV iron preparations may have been more appropriate. Some patients in the IV iron group (especially outpatients prescribed SFO) did not receive the required iron dose of 1000 mg or more. However, FCM maintained longer iron storage in the patients, especially at the appropriate total cumulative dose (1000 mg or 1500 mg) per the package insert. Underdiagnosis of IDA via serum ferritin and/or TSAT was observed in all disease areas and should be used more often to diagnose and treat patients appropriately. Post-treatment assessment rates to identify insufficient iron storage were also very low. Increasing post-treatment assessment rates can identify patients who may have failed initial treatment and need a change in the treatment method.

### Supplementary Information

Below is the link to the electronic supplementary material.

Supplementary file1 (DOCX 164 KB)

## Data Availability

The data that support the findings of this study (deidentified data regarding diseases, treatment, medication, tests, and other data) are available for purchase from Medical Data Vision Co., Ltd (MDV, Tokyo, Japan). Restrictions apply to the availability of these data, which were used under license for this study. For inquiries about access to the dataset used in this study, please contact MDV (website: https://en.mdv.co.jp/; email: ebm_sales@mdv.co.jp).
