# Supplementary material for: Clinical management of iron deficiency anemia in Japan: iron prescription patterns, treatment effectiveness, and assessments
Source: Int J Hematol. 2024 May 28;120(2):167–78. doi: 10.1007/s12185-024-03801-4 (PMC11284196; doi:10.1007/s12185-024-03801-4)
Supplement: Supplementary file 1 — Supplementary file1 (DOCX 164 KB) [file 12185_2024_3801_MOESM1_ESM.docx]

**Supplementary information**

**Table S1. Codes defined as treatment.**

| **Purpose** | **Category of treatment** | | **Codes** | | **Remarks** |
| --- | --- | --- | --- | --- | --- |
|  |  |  | **Type of codes** | **Details** |  |
| Inclusion criteria | Iron preparation | FCM | EphMRA-ATC | B3A1^*^ | The EphMRA-ATC code marked with "^*^" was included only if the Japanese-specific code 622676601 applied. |
|  |  | SFO | EphMRA-ATC | B3A1^†^ | The EphMRA-ATC code marked with "^†^" was included only if the Japanese-specific code 620005208 applied. |
|  |  | Oral iron | EphMRA-ATC | B3A1^‡^, V3G2^§^ | The EphMRA-ATC code marked with "^‡^" was included only if the route of administration was oral. The EphMRA-ATC code marked with "^§^" was included only if the Japanese-specific code 622306901 (Ferric citrate hydrate) applied. However, subjects are excluded in this definition if the standard disease code 8841364 (Hyperphosphatemia (including suspicion)) was also coded in the same month, because Ferric citrate hydrate is indicated for "hyperphosphatemia" and "iron deficiency anemia." |
| Concomitant treatment or exclusion criteria in the sensitivity analysis | Blood transfusion | | Japanese-specific code | 621772001, 621772101, 621772801, 621772901, 622191101, 622191201, 622191301, 622191401, 622191701, 622191801, 622191901, 622192001, 620004679, 620004680, 620004744, 620004745, 622190901, 622191001, 622191501, 622191601 | - |
| Concomitant treatment or exclusion criteria in the sensitivity analysis | Autologous blood donation | | Medical procedure code | K920^\|\|^ | The medical procedure code marked with "^\|\|^" was included only if any of the following Japanese-specific codes applied: 150327510, 150327610, 150327710, 150327810 |
| Exclusion criteria | Dialysis | | Medical procedure code | B001^¶^, C102, C102-2, C154, C155, C156, J038, J038-2, J042 | The medical procedure code marked with"^¶^" was included only if the Japanese-specific code 1130002510 applied. |

Abbreviations: EphMRA-ATC, the European pharmaceutical market research association anatomical therapeutic chemical; FCM, ferric carboxymaltose; SFO, saccharated ferric oxide.

**Table S2. Codes defined as disease area.**

A list of codes to be used for comorbidity or subgroup of disease area is shown below.

| **Category of disease area** | | **Codes** | | **Remarks** |
| --- | --- | --- | --- | --- |
|  |  | **Type of codes** | **Details** |  |
| **Gynecology** | Hypermenorrhea | ICD-10 | N92.x | - |
|  | Abnormal uterine bleeding | ICD-10 | N93.x | - |
|  | Fibroids | ICD-10 | D25.x | - |
|  | Adenomyosis or Endometriosis | ICD-10 | N80.x | - |
|  | Uterine fibroid-related surgery | Medical procedure codes | K871, K872, K872-2, K872-3, K872-4, K872-5, K873, K876, K876-2, K877, K877-2, K878, K878-2 | - |
| **Obstetrics** | Pregnancy | ICD-10 | O20.x, O99.0^*^ | The ICD-10 code marked with "^*^" was included only if any of the following standard disease codes applied: 2800013, 8838634 |
|  | Childbirth and puerperium | ICD-10 | O03.1, O03.6, O04.1, O04.6, O05.1, O05.6, O06.1, O06.6, O07.1, O07.6, O08.1, O44.1, O45.x, O46.x, O71.x, O72.x, O99.0^†^, O99.1^†^ | ICD-10 codes marked with “^†^” were included only if any of the following standard disease codes applied: 6663003, 8834085, 8847341, 8851088 |
|  |  | Medical procedure code | K898 | - |
| **IBD** | CD | ICD-10 | K50.x | - |
|  | UC | ICD-10 | K51.x | - |
| **Gastrointestinal bleeding  (excluding IBD)** | | ICD-10 | K22.8^‡^, K25.x^‡^, K26.x^‡^, K28.4, K29.0, K57.x^‡^, K62.5, K92.0, K92.1, K92.2 | ICD-10 codes marked with "^‡^" were included only if any of the following standard disease codes applied: 5308005, 5319011, 5350009, 8834632, 8834641, 8845122, 8845123, 8845124, 8845130, 8845131, 8845742, 8845749, 8845763, 8845800, 8845806, 8845814, 8847788 |
|  |  | Medical procedure code | K646, K654, K722 | - |
| **Gastrointestinal malabsorption (excluding IBD)** | | ICD-10 | K90.x, K91.x^§^ | The ICD-10 code marked with “^§^” was included only if any of the following standard disease codes applied: 2819001, 5644004, 8830480, 8835330, 8841646 |
|  |  | Medical procedure code | K654-2, K654-3, K654-4, K655, K655-2, K655-3, K655-4, K655-5, K657, K657-2, K716^\|\|^, K716-2^\|\|^ | Medical procedure codes marked with "^\|\|^" were included only if any of the following Japanese-specific codes applied: 150180650, 150181210, 150271950 |
| **Heart failure** | | ICD-10 | I09.9, I11.0, I13.x, I50.x | - |
| **Cancer** | | ICD-10 | C00-C97 | Subjects were excluded in the case of having the same standard disease code more than continuous 365 days before the index date. |
| **NDD-CKD** | | ICD-10 | E10.2, E11.2, E12.2, E13.2, E14.2, I12.x, I13.x,  N03.x, N05.x, N06.x, N07.x, N08.x, N18.x, N19.x | - |

Abbreviations: CD, Crohn's disease; IBD, inflammatory bowel disease; ICD-10, International Classification of Diseases, 10th Revision; NDD-CKD, non-dialysis dependent chronic kidney disease; UC, ulcerative colitis.

**Table S3. Codes defined as IDA-related tests.**

A list of codes to be used for the proportion of patients tested for diagnostic and post-treatment assessments of IDA is shown below.

| **Category of  IDA-related laboratory tests** | **Codes** | | **Remarks** |
| --- | --- | --- | --- |
|  | **Type of codes** | **Details** |  |
| Hb | Japanese-specific code | 160008010 | - |
| Serum iron | Japanese-specific code | 160022110 | - |
| Serum ferritin | Japanese-specific code | 160036810, 160192510 | - |
| TSAT | Japanese-specific code | 160022110^*^, 160023610^†^, 160023710^†^ | Subjects were included in the case of having the serum ferritin code marked with "^*^" on the same day as TSAT codes marked with "^†^" (any of the two marked). |

Abbreviations: Hb, hemoglobin; IDA, iron deficiency anemia; TSAT, transferrin saturation.

**Table S4. Iron prescriptions by disease area.**

|  | Total | | Gynecology | | Obstetrics | | IBD | | Gastrointestinal bleeding  or malabsorption  (excluding IBD) | | Heart failure | | Cancer | | NDD-CKD | |
| --- | --- | --- | --- | --- | --- | --- | --- | --- | --- | --- | --- | --- | --- | --- | --- | --- |
| Total | 368,945 | | 46,339 | | 47,856 | | 6,933 | | 46,496 | | 79,513 | | 92,566 | | 49,242 | |
| IV iron group*, n (%)* | 110,352 | (29.9) | 12,010 | (25.9) | 12,787 | (26.7) | 2,684 | (38.7) | 25,015 | (53.8) | 18,847 | (23.7) | 27,761 | (30.0) | 11,248 | (22.8) |
| FCM* | 7,145 | (6.5) | 2,076 | (17.3) | 858 | (6.7) | 607 | (22.6) | 917 | (3.7) | 803 | (4.3) | 1,503 | (5.4) | 381 | (3.4) |
| SFO* | 103,207 | (93.5) | 9,934 | (82.7) | 11,929 | (93.3) | 2,077 | (77.4) | 24,098 | (96.3) | 18,044 | (95.7) | 26,258 | (94.6) | 10,867 | (96.6) |
| Oral iron group*, n (%)* | 258,593 | (70.1) | 34,329 | (74.1) | 35,069 | (73.3) | 4,249 | (61.3) | 21,481 | (46.2) | 60,666 | (76.3) | 64,805 | (70.0) | 37,994 | (77.2) |

Abbreviations: FCM, ferric carboxymaltose; IBD, inflammatory bowel disease; IV, intravenous; NDD-CKD, non-dialysis dependent chronic kidney disease; SFO, saccharated ferric oxide.

*The denominator of proportion is the total number of patients prescribed IV iron.

**Table S5. Laboratory values and changes after STP.**

|  |  | **FCM** | | | |  | **SFO** | | | |  | **Oral iron** | | | |
| --- | --- | --- | --- | --- | --- | --- | --- | --- | --- | --- | --- | --- | --- | --- | --- |
|  |  | Laboratory values | | Changes* | |  | Laboratory values | | Changes* | |  | Laboratory values | | Changes* | |
|  |  | *n* | Median  (IQR) | *n* | Median  (IQR) |  | *n* | Median  (IQR) | *n* | Median  (IQR) |  | *n* | Median  (IQR) | *n* | Median  (IQR) |
| **Hb** | |  |  |  |  |  |  |  |  |  |  |  |  |  |  |
| Baseline | | 876 | 8.10  (7.10–9.10) | - | - |  | 11,034 | 8.70  (7.60–10.00) | - | - |  | 38,692 | 9.70  (8.60–10.80) | - | - |
| after  STP | Week 2 | 526 | 9.70  (8.70–10.60) | 519 | 1.70  (0.60–2.80) |  | 7,767 | 9.90  (8.80–11.00) | 7,635 | 1.00  (0.10–2.10) |  | 18,054 | 10.00  (8.90–11.10) | 17,179 | 0.40  (-0.40–1.30) |
|  | Week 4 | 475 | 10.90  (9.70–12.00) | 466 | 2.60  (1.20–4.10) |  | 6,118 | 10.80  (9.70–12.00) | 5,945 | 1.90  (0.70–3.30) |  | 18,707 | 10.80  (9.60–11.80) | 17,326 | 0.90  (-0.10–2.10) |
|  | Week 8 | 334 | 11.50  (10.20–12.60) | 329 | 3.10  (1.50–4.90) |  | 4,346 | 11.30  (10.00–12.50) | 4,182 | 2.35  (1.00–3.80) |  | 15,557 | 11.30  (10.00–12.40) | 14,120 | 1.40  (0.20–2.75) |
|  | Week 12 | 242 | 11.80  (10.20–12.90) | 236 | 3.20  (1.70–4.80) |  | 3,449 | 11.60  (10.30–12.90) | 3,288 | 2.60  （1.10–4.20) |  | 12,169 | 11.50  (10.30–12.80) | 10,999 | 1.70  (0.40–3.20) |
| **Serum ferritin** | |  |  |  |  |  |  |  |  |  |  |  |  |  |  |
| Baseline | | 267 | 17.80  (7.00–62.40) | - | - |  | 2,810 | 29.50  (11.00–94.00) | - | - |  | 9,344 | 21.10  (8.10–76.00) | - | - |
| after  STP | Week 2 | 75 | 282.00  (188.60–495.00) | 45 | 268.90  (155.40–478.00) |  | 832 | 232.40  (91.90–501.00) | 560 | 155.00  (45.10–383.15) |  | 1,958 | 58.20  (27.96–154.10) | 1,160 | 8.00  (-13.00–26.55) |
|  | Week 4 | 104 | 196.40  (68.15–329.10) | 71 | 136.60  (39.10–330.00) |  | 783 | 91.50  (37.60–261.00) | 526 | 50.70  (15.00–172.00) |  | 3,016 | 41.65  (23.00–88.00) | 1,953 | 16.20  (1.80–34.20) |
|  | Week 8 | 53 | 106.00  (40.00–279.10) | 35 | 62.00  (20.00–250.00) |  | 568 | 59.75  (24.75–162.00) | 393 | 24.00  (3.30–96.80) |  | 3,005 | 39.00  (21.00–80.30) | 1,873 | 17.00  (3.20–36.10) |
|  | Week 12 | 42 | 83.20  (30.30–217.20) | 27 | 68.40  (7.80–215.20) |  | 460 | 48.60  (20.65–148.00) | 298 | 20.15  (4.00–63.00) |  | 2,542 | 38.53  (20.00–78.00) | 1,618 | 18.00  (4.00–37.20) |
| **TSAT** | |  |  |  |  |  |  |  |  |  |  |  |  |  |  |
| Baseline | | 307 | 6.40  (3.28–14.84) | - | - |  | 2,984 | 8.62  (4.24–19.32) | - | - |  | 9,332 | 8.19  (4.46–16.54) | - | - |
| after  STP | Week 2 | 86 | 23.06  (15.28–33.04) | 56 | 15.59  (8.71–24.25) |  | 864 | 19.39  (12.35–30.09) | 623 | 8.37  (1.12–18.70) |  | 1,855 | 19.33  (11.26–31.67) | 1,130 | 5.73  (0.06–15.93) |
|  | Week 4 | 101 | 23.31  (16.81–32.32) | 74 | 16.15  (4.21–25.52) |  | 772 | 20.68  (12.38–31.20) | 557 | 8.22  (1.25–21.03) |  | 2,688 | 21.26  (13.15–33.84) | 1,804 | 10.17  (2.47–23.41) |
|  | Week 8 | 58 | 22.60  (16.15–32.37) | 43 | 13.03  (5.36–21.68) |  | 535 | 20.75  (11.46–31.14) | 383 | 9.56  (1.59–21.46) |  | 2,577 | 22.56  (14.07–33.47) | 1,727 | 11.49  (3.45–23.07) |
|  | Week 12 | 43 | 23.39  (13.04–30.45) | 34 | 16.26  (2.86–24.01) |  | 458 | 22.00  (12.50–31.58) | 339 | 9.36  (1.96–19.46) |  | 2,158 | 23.28  (14.84–33.22) | 1,447 | 12.21  (3.95–23.14) |

Abbreviations: FCM, ferric carboxymaltose; Hb, hemoglobin; IQR, interquartile range; SFO, saccharated ferric oxide; STP, The start date of Treatment Period; TSAT, transferrin saturation.

^*^Patients who have the values of the baseline and the values of each assessment time point are evaluated for "Changes."

**Table S6. Patient characteristics in the inpatient and outpatient.**

|  | Inpatient | | | | | | | | | Outpatient | | | | | |
| --- | --- | --- | --- | --- | --- | --- | --- | --- | --- | --- | --- | --- | --- | --- | --- |
|  | FCM | | | | SFO | | | Oral iron | | FCM | | SFO | | Oral iron | |
|  | (*N* = 2,835) | | | | (*N* = 82,044) | | | (*N* = 123,818) | | (*N* = 4,602) | | (*N* = 16,604) | | (*N* = 235,729) | |
| **Female gender*, n (%)*** | 2,024 | | (71.4) | | 48,548 | (59.2) | | 84,068 | (67.9) | 3,652 | (79.4) | 12,438 | (74.9) | 168,094 | (71.3) |
| **Age (years)** |  | |  | |  |  | |  |  |  |  |  |  |  |  |
| Median (IQR) | 57.0 (36.0–79.0) | | | | 72.0 (48.0–83.0) | | | 77.0 (45.0–86.0) | | 49.0 (42.0–68.0) | | 57.0 (43.0–75.0) | | 52.0 (37.0–76.0) | |
| < 20 years, *n (%)* | 25 | | (0.9) | | 742 | (0.9) | | 2,267 | (1.8) | 80 | (1.7) | 367 | (2.2) | 14,723 | (6.2) |
| ≥ 20 and < 45 years, *n (%)* | 1,084 | | (38.2) | | 17,889 | (21.8) | | 28,310 | (22.9) | 1,459 | (31.7) | 4,202 | (25.3) | 73,629 | (31.2) |
| ≥ 45 and < 55 years, *n (%)* | 273 | | (9.6) | | 6,045 | (7.4) | | 5,247 | (4.2) | 1,455 | (31.6) | 3,472 | (20.9) | 35,201 | (14.9) |
| ≥ 55 and < 65 years, *n (%)* | 169 | | (6.0) | | 6,392 | (7.8) | | 5,598 | (4.5) | 321 | (7.0) | 1,451 | (8.7) | 14,040 | (6.0) |
| ≥ 65 years, *n (%)* | 1,284 | | (45.3) | | 50,976 | (62.1) | | 82,396 | (66.5) | 1,287 | (28.0) | 7,112 | (42.8) | 98,136 | (41.6) |
| **Weight (kg)** |  | |  | |  |  | |  |  |  |  |  |  |  |  |
| Median (IQR) | 55.10 (47.80–64.00) | | | | 54.90 (46.70–63.60) | | | 52.60 (44.20–61.50) | | 55.35 (48.00–64.60) | | 56.00 (48.00–65.00) | | 53.60 (44.60–63.00) | |
| *n (%)* | 2,642 | | (93.2) | | 76,098 | (92.8) | | 111,764 | (90.3) | 426 | (9.3) | 2,054 | (12.4) | 52,416 | (22.2) |
| < 25 kg | 2 | | (0.1) | | 170 | (0.2) | | 1,558 | (1.4) | 1 | (0.2) | 5 | (0.2) | 5,349 | (10.2) |
| ≥ 25 and < 35 kg | 68 | | (2.6) | | 2,414 | (3.2) | | 4,865 | (4.4) | 4 | (0.9) | 45 | (2.2) | 988 | (1.9) |
| ≥ 35 and < 70 kg | 2,221 | | (84.1) | | 63,189 | (83.0) | | 93,305 | (83.5) | 354 | (83.1) | 1,676 | (81.6) | 39,332 | (75.0) |
| ≥ 70 kg | 351 | | (13.3) | | 10,325 | (13.6) | | 12,036 | (10.8) | 67 | (15.7) | 328 | (16.0) | 6,747 | (12.9) |
| **Blood transfusion within 42 days before the Index date** | | | | | | | | | | | | | | | |
| *n (%)* | 638 | | (22.5) | | 17,658 | (21.5) | | 13,918 | (11.2) | 202 | (4.4) | 508 | (3.1) | 10,208 | (4.3) |
| > 400 mL | 441 | | (69.1) | | 12,075 | (68.4) | | 8422 | (60.5) | 131 | (64.9) | 314 | (61.8) | 6,606 | (64.7) |
| **Blood transfusion in the Treatment Period** | | | | | | | | | | | | | | | |
| *n (%)* | 342 | | (12.1) | | 19,801 | (24.1) | | 13,729 | (11.1) | 148 | (3.2) | 1,102 | (6.6) | 14,121 | (6.0) |
| > 400 mL | 188 | | (55.0) | | 12,519 | (63.2) | | 7,469 | (54.4) | 82 | (55.4) | 676 | (61.3) | 8,726 | (61.8) |
| **Autologous blood donation in the Treatment Period** | | | | | | | | | | | | | | | |
| *n (%)* | 8 | | (0.3) | | 483 | (0.6) | | 792 | (0.6) | 278 | (6.0) | 3,643 | (21.9) | 10,154 | (4.3) |
| > 400 mL | 0 | | (0.0) | | 206 | (42.7) | | 159 | (20.1) | 40 | (14.4) | 1,695 | (46.5) | 4,390 | (43.2) |
| **Hb (g/dL)** |  | |  | |  |  | |  |  |  |  |  |  |  |  |
| Median (IQR) | 8.20 (7.40–8.90) | | | | 8.70 (7.60–9.90) | | | 9.50 (8.50–10.60) | | 7.90 (6.90–9.20) | | 8.90 (7.40–10.60) | | 9.80 (8.70–10.80) | |
| *n (%)* | 487 | | (17.2) | | 9,616 | (11.7) | | 14,569 | (11.8) | 389 | (8.5) | 1,418 | (8.5) | 24,123 | (10.2) |
| < 8 g/dL | 206 | | (42.3) | | 3,156 | (32.8) | | 2,182 | (15.0) | 201 | (51.7) | 484 | (34.1) | 3,361 | (13.9) |
| ≥ 8 g/dL | 281 | | (57.7) | | 6,460 | (67.2) | | 12,387 | (85.0) | 188 | (48.3) | 934 | (65.9) | 20,762 | (86.1) |
| < 10 g/dL | 450 | | (92.4) | | 7,307 | (76.0) | | 9,308 | (63.9) | 328 | (84.3) | 955 | (67.3) | 13,188 | (54.7) |
| ≥ 10 g/dL | 37 | | (7.6) | | 2,309 | (24.0) | | 5,261 | (36.1) | 61 | (15.7) | 463 | (32.7) | 10,935 | (45.3) |
| **Serum ferritin (ng/mL)** | | | | | | | | | | | | | | | |
| Median (IQR) | 36.10 (14.50–130.25) | | | | 36.00 (13.80–114.00) | | | 66.90 (26.00–198.10) | | 8.90 (4.30–22.50) | | 11.70 (5.00–29.20) | | 14.50 (6.80–45.50) | |
| *n (%)* | 132 | | (4.7) | | 2,339 | (2.9) | | 2,317 | (1.9) | 135 | (2.9) | 471 | (2.8) | 7,027 | (3.0) |
| < 12 ng/mL | 25 | | (18.9) | | 504 | (21.5) | | 210 | (9.1) | 79 | (58.5) | 237 | (50.3) | 3,043 | (43.3) |
| ≥ 12 ng/mL | 107 | | (81.1) | | 1,835 | (78.5) | | 2,107 | (90.9) | 56 | (41.5) | 234 | (49.7) | 3,984 | (56.7) |
| **Serum iron (µg/dL)** | | | | | | | | | | | | | | | |
| Median (IQR) | 22.00 (13.00–39.00) | | | | 24.00 (14.00–47.00) | | | 29.00 (18.00–48.00) | | 19.00 (11.00–39.00) | | 20.00 (12.00–43.00) | | 26.00 (16.00–48.00) | |
| *n (%)* | 194 | | (6.8) | | 4,118 | (5.0) | | 3,831 | (3.1) | 197 | (4.3) | 730 | (4.4) | 10,874 | (4.6) |
| **TSAT (%)** |  | |  | |  |  | |  |  |  |  |  |  |  |  |
| Median (IQR) | 8.43 (5.11–18.18) | | | | 9.29 (4.66–19.82) | | | 11.49 (6.54–20.10) | | 4.95 (2.68–11.89) | | 5.29 (2.72–13.79) | | 7.14 (4.02–14.70) | |
| *n (%)* | 166 | | (5.9) | | 2,521 | (3.1) | | 2,421 | (2.0) | 141 | (3.1) | 463 | (2.8) | 6,911 | (2.9) |
| < 20% | 127 | | (76.5) | | 1,892 | (75.0) | | 1,809 | (74.7) | 121 | (85.8) | 370 | (79.9) | 5,707 | (82.6) |
| ≥ 20% | 39 | | (23.5) | | 629 | (25.0) | | 612 | (25.3) | 20 | (14.2) | 93 | (20.1) | 1,204 | (17.4) |
| **CRP (mg/dL)** |  | |  | |  |  | |  |  |  |  |  |  |  |  |
| *n (%)* | 456 | | (16.1) | | 8,711 | (10.6) | | 12,830 | (10.4) | 238 | (5.2) | 863 | (5.2) | 14,149 | (6.0) |
| ≤ 1.0 mg/dL | 218 | | (47.8) | | 4,323 | (49.6) | | 5,623 | (43.8) | 201 | (84.5) | 684 | (79.3) | 10,771 | (76.1) |
| > 1.0 mg/dL | 238 | | (52.2) | | 4,388 | (50.4) | | 7,207 | (56.2) | 37 | (15.5) | 179 | (20.7) | 3,378 | (23.9) |
| **Inpatient or Outpatient*, n (%)*** | | | | | | | | | | | | | | | |
| Inpatient | 2,835 | | (100.0) | | 82,044 | (100.0) | | 123,818 | (100.0) | 0 | (0.0) | 0 | (0.0) | 0 | (0.0) |
| Outpatient | 0 | | (0.0) | | 0 | (0.0) | | 0 | (0.0) | 4,602 | (100.0) | 16,604 | (100.0) | 235,729 | (100.0) |
| **Comorbidities*, *n (%)*** | | | | | | | | | | | | | | | |
| Gynecology | 354 | | (12.5) | | 6,348 | (7.7) | | 4,723 | (3.8) | 1,722 | (37.4) | 3,586 | (21.6) | 29,606 | (12.6) |
| Heavy menstrual bleeding^†^ | 77 | | (21.8) | | 1,346 | (21.2) | | 630 | (13.3) | 615 | (35.7) | 1,416 | (39.5) | 7,969 | (26.9) |
| Abnormal uterine bleeding^†^ | 56 | | (15.8) | | 1,213 | (19.1) | | 739 | (15.6) | 252 | (14.6) | 583 | (16.3) | 4,090 | (13.8) |
| Fibroids^†^ | 248 | | (70.1) | | 4,373 | (68.9) | | 3,155 | (66.8) | 1,225 | (71.1) | 2,577 | (71.9) | 21,553 | (72.8) |
| Adenomyosis or Endometriosis^†^ | | 91 | | (25.7) | 1,572 | (24.8) | | 1,148 | (24.3) | 532 | (30.9) | 1,028 | (28.7) | 6,916 | (23.4) |
| Uterine fibroid-related surgery^†^ | 164 | | (46.3) | | 2,717 | (42.8) | | 1,740 | (36.8) | 21 | (1.2) | 31 | (0.9) | 783 | (2.6) |
| Obstetrics | 718 | | (25.3) | | 11,199 | (13.6) | | 17,084 | (13.8) | 140 | (3.0) | 730 | (4.4) | 17,985 | (7.6) |
| Pregnancy^†^ | 188 | | (26.2) | | 3,260 | (29.1) | | 4,606 | (27.0) | 89 | (63.6) | 510 | (69.9) | 13,788 | (76.7) |
| Childbirth and the puerperium^†^ | 670 | | (93.3) | | 10,428 | (93.1) | | 14,680 | (85.9) | 73 | (52.1) | 328 | (44.9) | 5,464 | (30.4) |
| IBD | 104 | | (3.7) | | 1,464 | (1.8) | | 471 | (0.4) | 503 | (10.9) | 613 | (3.7) | 3,778 | (1.6) |
| UC^†^ | 67 | | (64.4) | | 1,072 | (73.2) | | 352 | (74.7) | 252 | (50.1) | 361 | (58.9) | 2,811 | (74.4) |
| CD^†^ | 39 | | (37.5) | | 434 | (29.6) | | 133 | (28.2) | 280 | (55.7) | 272 | (44.4) | 1,081 | (28.6) |
| Gastrointestinal bleeding  (excluding IBD) | 542 | | (19.1) | | 21,894 | (26.7) | | 7,216 | (5.8) | 310 | (6.7) | 968 | (5.8) | 12,927 | (5.5) |
| Gastrointestinal malabsorption  (excluding IBD) | 44 | | (1.6) | | 1,359 | (1.7) | | 493 | (0.4) | 31 | (0.7) | 122 | (0.7) | 998 | (0.4) |
| Heart failure | 433 | | (15.3) | | 15,881 | (19.4) | | 25,375 | (20.5) | 370 | (8.0) | 2,163 | (13.0) | 35,291 | (15.0) |
| Cancer | 725 | | (25.6) | | 23,285 | (28.4) | | 20,220 | (16.3) | 778 | (16.9) | 2,973 | (17.9) | 44,585 | (18.9) |
| NDD-CKD | 177 | | (6.2) | | 8,911 | (10.9) | | 15,142 | (12.2) | 204 | (4.4) | 1,956 | (11.8) | 22,852 | (9.7) |
| **Total iron dose in the Treatment Period (mg)** | | | | | | |  | | | | | | | | |
| Median (IQR) | 500.00  (500.00–500.00) | | | | 320.00  (160.00–560.00) | | | 1,400.00  (700.00–2,700.00) | | 500.00  (500.00–1,000.00) | | 120.00  (80.00–240.00) | | 4,000.00  (2,100.00–8,400.00) | |
| **Total IV iron dose in the Treatment Period by prescription dose (mg)** | | | | | | | | | | | | | | | |
| ≤500 mg*, n (%)* | 2,195 | | (77.4) | | 60,247 | (73.4) | | - | - | 23,69 | (51.5) | 14,326 | (86.3) | - | - |
| > 500 and ≤ 1,000 mg*, n (%)* | 441 | | (15.6) | | 15,928 | (19.4) | | - | - | 1,099 | (23.9) | 1,554 | (9.4) | - | - |
| > 1,000 and ≤ 1,500 mg*, n (%)* | 180 | | (6.3) | | 3,560 | (4.3) | | - | - | 1,040 | (22.6) | 426 | (2.6) | - | - |
| > 1,500 mg*, n (%)* | 19 | | (0.7) | | 2,309 | (2.8) | | - | - | 94 | (2.0) | 298 | (1.8) | - | - |
| **Duration in the Treatment Period (days)** | | | | | | |  | | | | | | | | |
| Median (IQR) | 1.0 (1.0–1.0) | | | | 5.0 (3.0–8.0) | | | 14.0 (7.0–26.0) | | 1.0 (1.0–15.0) | | 3.0 (1.0–20.0) | | 46.0 (27.0–91.0) | |
| **Mean iron dose per a day in the Treatment Period (mg)** | | | | | | |  | | | | | | | | |
| Median (IQR) | 500.00 (500.00–500.00) | | | | 80.00 (40.00–80.00) | | | 100.00 (88.89–107.69) | | 500.00 (100.00–500.00) | | 40.00 (13.33–80.00) | | 100.00 (62.40–105.00) | |
| **Combined with oral iron in IV iron groups from 42 days before the Index date to ETP** | | | | | | | | | | | | | | | |
| *n (%)* | 709 | | | (25.0) | 23,337 | (28.4) | | - | | 1,343 | (29.2) | 8,843 | (53.3) | - | |

Abbreviations: CD, Crohn's disease; CRP, C-reactive protein; ETP, The end of Treatment Period; FCM, ferric carboxymaltose; Hb, hemoglobin; IBD, inflammatory bowel disease; IQR, interquartile range; IV, intravenous; NDD-CKD, non-dialysis dependent chronic kidney disease; SFO, saccharated ferric oxide; TSAT, transferrin saturation; UC, ulcerative colitis.

^*^Multiple choice allowed.

^†^The denominator of n (%) is the patients who applied the superordinate disease area in each iron group.

**Table S7a. Proportion of patients tested for diagnosis of IDA in each disease area.**

|  |  | **FCM** | | **SFO** | | **Oral iron** | |
| --- | --- | --- | --- | --- | --- | --- | --- |
| Gynecology*, n (%)* | Total | 2,076 | | 9,934 | | 34,329 | |
|  | Hb | 1,553 | (74.8) | 8,509 | (85.7) | 22,233 | (64.8) |
|  | Serum iron | 719 | (34.6) | 2,490 | (25.1) | 7,673 | (22.4) |
|  | Serum ferritin | 542 | (26.1) | 1,625 | (16.4) | 5,699 | (16.6) |
|  | TSAT | 442 | (21.3) | 1,450 | (14.6) | 4,350 | (12.7) |
| Obstetrics*, n (%)* | Total | 858 | | 11,929 | | 35,069 | |
|  | Hb | 670 | (78.1) | 9,905 | (83.0) | 18,757 | -(53.5) |
|  | Serum iron | 47 | (5.5) | 841 | (7.1) | 996 | (2.8) |
|  | Serum ferritin | 29 | (3.4) | 306 | (2.6) | 392 | (1.1) |
|  | TSAT | 25 | (2.9) | 248 | (2.1) | 261 | (0.7) |
| IBD*, n (%)* | Total | 607 | | 2,077 | | 4,249 | |
|  | Hb | 472 | (77.8) | 1,772 | (85.3) | 3,128 | (73.6) |
|  | Serum iron | 401 | (66.1) | 1,262 | (60.8) | 2,191 | (51.6) |
|  | Serum ferritin | 333 | (54.9) | 1,038 | (50.0) | 1,799 | (42.3) |
|  | TSAT | 151 | (24.9) | 632 | (30.4) | 1,177 | (27.7) |
| Gastrointestinal bleeding | Total | 917 | | 24,098 | | 21,481 | |
| or malabsorption | Hb | 831 | (90.6) | 23,504 | (97.5) | 19,281 | (89.8) |
| (excluding IBD)*, n (%)* | Serum iron | 526 | (57.4) | 13,092 | (54.3) | 11,357 | (52.9) |
|  | Serum ferritin | 401 | (43.7) | 10,759 | (44.6) | 9,760 | (45.4) |
|  | TSAT | 362 | (39.5) | 8,669 | (36.0) | 7,610 | (35.4) |
| Heart failure*, n (%)* | Total | 803 | | 18,044 | | 60,666 | |
|  | Hb | 711 | (88.5) | 17,139 | (95.0) | 51,863 | (85.5) |
|  | Serum iron | 557 | (69.4) | 11,300 | (62.6) | 30,942 | (51.0) |
|  | Serum ferritin | 515 | (64.1) | 10,041 | (55.6) | 28,560 | (47.1) |
|  | TSAT | 428 | (53.3) | 7,878 | (43.7) | 22,025 | (36.3) |
| Cancer*, n (%)* | Total | 1,503 | | 26,258 | | 64,805 | |
|  | Hb | 1,323 | (88.0) | 24,946 | (95.0) | 54,343 | (83.9) |
|  | Serum iron | 749 | (49.8) | 14,203 | (54.1) | 26,705 | (41.2) |
|  | Serum ferritin | 543 | (36.1) | 10,294 | (39.2) | 20,329 | (31.4) |
|  | TSAT | 461 | (30.7) | 8,573 | (32.6) | 16,038 | (24.7) |
| NDD-CKD*, n (%)* | Total | 381 | | 10867 | | 37994 | |
|  | Hb | 333 | (87.4) | 9,923 | (91.3) | 31,456 | (82.8) |
|  | Serum iron | 268 | (70.3) | 6,467 | (59.5) | 20,192 | (53.1) |
|  | Serum ferritin | 248 | (65.1) | 5,797 | (53.3) | 19,389 | (51.0) |
|  | TSAT | 215 | (56.4) | 4,696 | (43.2) | 15,305 | (40.3) |

**Table S7b. Proportion of patients tested for post-treatment assessments of IDA in each disease area.**

|  |  | **FCM** | | **SFO** | | **Oral iron** | |
| --- | --- | --- | --- | --- | --- | --- | --- |
| Gynecology*, n (%)* | Total | 2,076 | | 9,934 | | 34,329 | |
|  | Hb | 1,322 | (63.7) | 7,766 | (78.2) | 11,936 | (34.8) |
|  | Serum iron | 437 | (21.1) | 1,429 | (14.4) | 2,639 | (7.7) |
|  | Serum ferritin | 332 | (16.0) | 984 | (9.9) | 1,982 | (5.8) |
|  | TSAT | 227 | (10.9) | 774 | (7.8) | 1,414 | (4.1) |
| Obstetrics*, n (%)* | Total | 858 | | 11,929 | | 35,069 | |
|  | Hb | 603 | (70.3) | 9,290 | (77.9) | 8,712 | (24.8) |
|  | Serum iron | 81 | (9.4) | 609 | (5.1) | 463 | (1.3) |
|  | Serum ferritin | 67 | (7.8) | 325 | (2.7) | 246 | (0.7) |
|  | TSAT | 54 | (6.3) | 277 | (2.3) | 200 | (0.6) |
| IBD*, n (%)* | Total | 607 | | 2,077 | | 4,249 | |
|  | Hb | 409 | (67.4) | 1,594 | (76.7) | 2,245 | (52.8) |
|  | Serum iron | 326 | (53.7) | 951 | (45.8) | 1,674 | (39.4) |
|  | Serum ferritin | 257 | (42.3) | 721 | (34.7) | 1,315 | (30.9) |
|  | TSAT | 97 | (16.0) | 392 | (18.9) | 826 | (19.4) |
| Gastrointestinal bleeding | Total | 917 | | 24,098 | | 21,481 | |
| or malabsorption | Hb | 755 | (82.3) | 20,770 | (86.2) | 9,425 | (43.9) |
| (excluding IBD)*, n (%)* | Serum iron | 395 | (43.1) | 8,466 | (35.1) | 4,709 | (21.9) |
|  | Serum ferritin | 285 | (31.1) | 6,557 | (27.2) | 3,728 | (17.4) |
|  | TSAT | 235 | (25.6) | 4,878 | (20.2) | 2,805 | (13.1) |
| Heart failure*, n (%)* | Total | 803 | | 18,044 | | 60,666 | |
|  | Hb | 653 | (81.3) | 15,625 | (86.6) | 26,697 | (44.0) |
|  | Serum iron | 390 | (48.6) | 6,711 | (37.2) | 12,199 | (20.1) |
|  | Serum ferritin | 342 | (42.6) | 5,487 | (30.4) | 11,193 | (18.5) |
|  | TSAT | 261 | (32.5) | 4,044 | (22.4) | 7,904 | (13.0) |
| Cancer*, n (%)* | Total | 1,503 | | 26,258 | | 64,805 | |
|  | Hb | 1,228 | (81.7) | 22,681 | (86.4) | 35,096 | (54.2) |
|  | Serum iron | 471 | (31.3) | 8,087 | (30.8) | 11,014 | (17.0) |
|  | Serum ferritin | 309 | (20.6) | 5,072 | (19.3) | 7,861 | (12.1) |
|  | TSAT | 251 | (16.7) | 4,022 | (15.3) | 5,889 | (9.1) |
| NDD-CKD*, n (%)* | Total | 381 | | 10,867 | | 37,994 | |
|  | Hb | 295 | (77.4) | 8,522 | 78.4 | 17,128 | (45.1) |
|  | Serum iron | 189 | (49.6) | 3,920 | (36.1) | 9,265 | (24.4) |
|  | Serum ferritin | 171 | (44.9) | 3,368 | (31.0) | 9,012 | (23.7) |
|  | TSAT | 140 | (36.7) | 2,633 | (24.2) | 6,782 | (17.9) |

Abbreviations; FCM, ferric carboxymaltose; Hb, hemoglobin; IBD, inflammatory bowel disease; IDA, iron deficiency anemia; NDD-CKD, non-dialysis dependent chronic kidney disease; SFO, saccharated ferric oxide; TSAT, transferrin saturation

**Table S8a. Patient characteristics in each disease area (Gynecology, Obstetrics).**

|  | Gynecology | | | | | | Obstetrics | | | | | | |  |
| --- | --- | --- | --- | --- | --- | --- | --- | --- | --- | --- | --- | --- | --- | --- |
|  | FCM | | SFO | | Oral iron | | FCM | | SFO | | Oral iron | | |  |
|  | (*N* = 2,076) | | (*N* = 9,934) | | (*N* = 34,329) | | (*N* = 858) | | (*N* = 11,929) | | (*N* = 35,069) | | |  |
| **Female gender*, n (%)*** | 2,076 | (100.0) | 9,934 | (100.0) | 34,329 | (100.0) | 858 | (100.0) | 11,929 | (100.0) | 35,069 | | (100.0) | |
| **Age (years)** |  |  |  |  |  |  |  |  |  |  |  | |  | |
| Median (IQR) | 46.0 (41.0–49.0) | | 45.0 (39.0–50.0) | | 45.0 (39.0–49.0) | | 34.0 (30.0–37.0) | | 33.0 (30.0–37.0) | | 33.0 (29.0–37.0) | | |  |
| < 20 years, *n (%)* | 19 | (0.9) | 103 | (1.0) | 269 | (0.8) | 8 | (0.9) | 73 | (0.6) | 307 | | (0.9) | |
| ≥ 20 and < 45 years, *n (%)* | 863 | (41.6) | 4,611 | (46.4) | 16,248 | (47.3) | 840 | (97.9) | 11,778 | (98.7) | 34,557 | | (98.5) | |
| ≥ 45 and < 55 years, *n (%)* | 1,094 | (52.7) | 4,074 | (41.0) | 15,572 | (45.4) | 8 | (0.9) | 74 | (0.6) | 181 | | (0.5) | |
| ≥ 55 and < 65 years, *n (%)* | 57 | (2.7) | 457 | (4.6) | 895 | (2.6) | 1 | (0.1) | 2 | (0.0) | 6 | | (0.0) | |
| ≥ 65 years, *n (%)* | 43 | (2.1) | 689 | (6.9) | 1,345 | (3.9) | 1 | (0.1) | 2 | (0.0) | 18 | | (0.1) | |
| **Weight (kg)** |  |  |  |  |  |  |  |  |  |  |  | |  | |
| Median (IQR) | 56.00 (50.00–65.00) | | 56.80 (50.40–65.00) | | 56.60 (50.10–65.00) | | 60.10 (54.00–67.00) | | 61.10 (55.00–68.30) | | 61.00 (54.90–68.40) | | |  |
| *n (%)* | 436 | (21.0) | 6,421 | (64.6) | 7,243 | (21.1) | 703 | (81.9) | 10,502 | (88.0) | 18,339 | | (52.3) | |
| < 25 kg | 0 | (0.0) | 1 | (0.0) | 2 | (0.0) | 0 | (0.0) | 1 | (0.0) | 1 | | (0.0) | |
| ≥ 25 and < 35 kg | 0 | (0.0) | 30 | (0.5) | 40 | (0.6) | 2 | (0.3) | 6 | (0.1) | 3 | | (0.0) | |
| ≥ 35 and < 70 kg | 364 | (83.5) | 5,402 | (84.1) | 6,044 | (83.4) | 571 | (81.2) | 8,284 | (78.9) | 14,373 | | (78.4) | |
| ≥ 70 kg | 72 | (16.5) | 988 | (15.4) | 1,157 | (16.0) | 130 | (18.5) | 2,211 | (21.1) | 3,962 | | (21.6) | |
| **Blood transfusion within 42 days before the Index date** | | | | | | | | | | | |  | |  |
| *n (%)* | 107 | (5.2) | 881 | (8.9) | 854 | (2.5) | 80 | (9.3) | 697 | (5.8) | 543 | | (1.5) | |
| > 400 mL | 78 | (72.9) | 690 | (78.3) | 670 | (78.5) | 61 | (76.3) | 582 | (83.5) | 464 | | (85.5) | |
| **Blood transfusion in the Treatment Period** | | | | | | | | | | | |  | |  |
| *n (%)* | 69 | (3.3) | 917 | (9.2) | 736 | (2.1) | 35 | (4.1) | 626 | (5.2) | 365 | | (1.0) | |
| > 400 mL | 43 | (62.3) | 650 | (70.9) | 523 | (71.1) | 20 | (57.1) | 444 | (70.9) | 280 | | (76.7) | |
| **Autologous blood donation in the Treatment Period** | | | | | | | | | | | |  | |  |
| *n (%)* | 41 | (2.0) | 436 | (4.4) | 1,518 | (4.4) | 32 | (3.7) | 505 | (4.2) | 1,214 | | (3.5) | |
| > 400 mL | 10 | (24.4) | 123 | (28.2) | 468 | (30.8) | 7 | (21.9) | 249 | (49.3) | 610 | | (50.2) | |
| **Hb (g/dL)** |  |  |  |  |  |  |  |  |  |  |  | |  | |
| Median (IQR) | 7.70 (6.80–8.90) | | 8.80 (7.60–9.90) | | 9.90 (8.90–10.80) | | 8.25 (7.70–9.00) | | 9.20 (8.30–10.20) | | 9.70 (9.20–10.20) | | |  |
| *n (%)* | 182 | (8.8) | 867 | (8.7) | 2,870 | (8.4) | 106 | (12.4) | 1,230 | (10.3) | 3,226 | | (9.2) | |
| < 8 g/dL | 100 | (54.9) | 273 | (31.5) | 344 | (12.0) | 41 | (38.7) | 227 | (18.5) | 63 | | (2.0) | |
| ≥ 8 g/dL | 82 | (45.1) | 594 | (68.5) | 2,526 | (88.0) | 65 | (61.3) | 1,003 | (81.5) | 3,163 | | (98.0) | |
| < 10 g/dL | 165 | (90.7) | 656 | (75.7) | 1,509 | (52.6) | 96 | (90.6) | 872 | (70.9) | 2,085 | | (64.6) | |
| ≥ 10 g/dL | 17 | (9.3) | 211 | (24.3) | 1,361 | (47.4) | 10 | (9.4) | 358 | (29.1) | 1,141 | | (35.4) | |
| **Serum ferritin (ng/mL)** |  |  |  |  |  |  |  |  |  |  |  | |  | |
| Median (IQR) | 4.65 (2.00–19.90) | | 15.25 (6.50–36.15) | | 7.45 (4.40–19.30) | | 19.50 (14.80–85.00) | | 16.10 (8.93–21.60) | | 9.20 (5.90–16.00) | | |  |
| *n (%)* | 32 | (1.5) | 100 | (1.0) | 398 | (1.2) | 4 | (0.5) | 19 | (0.2) | 19 | | (0.1) | |
| < 12 ng/mL | 22 | (68.8) | 45 | (45.0) | 254 | (63.8) | 1 | (25.0) | 8 | (42.1) | 13 | | (68.4) | |
| ≥ 12 ng/mL | 10 | (31.3) | 55 | (55.0) | 144 | (36.2) | 3 | (75.0) | 11 | (57.9) | 6 | | (31.6) | |
| **Serum iron (µg/dL)** |  |  |  |  |  |  |  |  |  |  |  | |  | |
| Median (IQR) | 11.50 (9.00–23.50) | | 21.00 (11.00–49.00) | | 22.00 (13.00–45.00) | | 64.00 (45.00–70.00) | | 49.00 (32.00–103.00) | | 43.00 (31.00–60.50) | | |  |
| *n (%)* | 52 | (2.5) | 189 | (1.9) | 749 | (2.2) | 5 | (0.6) | 54 | (0.5) | 68 | | (0.2) | |
| **TSAT (%)** |  |  |  |  |  |  |  |  |  |  |  | |  | |
| Median (IQR) | 2.93 (1.71–7.88) | | 6.61 (2.88–20.37) | | 4.88 (2.99–11.68) | | 14.56 (13.11–21.67) | | 18.05 (8.74–29.67) | | 9.16 (7.89–14.89) | | |  |
| *n (%)* | 32 | (1.5) | 111 | (1.1) | 348 | (1.0) | 5 | (0.6) | 30 | (0.3) | 20 | | (0.1) | |
| < 20% | 29 | (90.6) | 80 | (72.1) | 295 | (84.8) | 3 | (60.0) | 16 | (53.3) | 18 | | (90.0) | |
| ≥ 20% | 3 | (9.4) | 31 | (27.9) | 53 | (15.2) | 2 | (40.0) | 14 | (46.7) | 2 | | (10.0) | |
| **CRP (mg/dL)** |  |  |  |  |  |  |  |  |  |  |  | |  | |
| *n (%)* | 103 | (5.0) | 577 | (5.8) | 1,248 | (3.6) | 87 | (10.1) | 952 | (8.0) | 1,654 | | (4.7) | |
| ≤ 1.0 mg/dL | 79 | (76.7) | 316 | (54.8) | 868 | (69.6) | 26 | (29.9) | 459 | (48.2) | 768 | | (46.4) | |
| > 1.0 mg/dL | 24 | (23.3) | 261 | (45.2) | 380 | (30.6) | 61 | (70.1) | 493 | (51.8) | 886 | | (53.6) | |
| **Inpatient or Outpatient*, n (%)*** |  |  |  |  |  |  |  |  |  |  |  | |  | |
| Inpatient | 354 | (17.1) | 6,348 | (63.9) | 4,723 | (13.8) | 718 | (83.7) | 11,199 | (93.9) | 17,084 | | (48.7) | |
| Outpatient | 1,722 | (82.9) | 3,586 | (36.1) | 29,606 | (86.2) | 140 | (16.3) | 730 | (6.1) | 17,985 | | (51.3) | |
| **"Inpatient or Outpatient" and "Hb ≥ 8 or < 8 g/dL", n (%)^‡^** | | | | | | | | | | | |  | |  |
| Inpatient and Hb < 8 g/dL | 14 | (7.7) | 151 | (17.4) | 32 | (1.1) | 40 | (37.7) | 223 | (18.1) | 30 | | (0.9) | |
| Inpatient and Hb ≥ 8 g/dL | 20 | (11.0) | 442 | (51.0) | 431 | (15.0) | 57 | (53.8) | 949 | (77.2) | 1,651 | | (51.2) | |
| Outpatient and Hb < 8 g/dL | 86 | (47.3) | 122 | (14.1) | 312 | (10.9) | 1 | (0.9) | 4 | (0.3) | 33 | | (1.0) | |
| Outpatient and Hb ≥ 8 g/dL | 62 | (34.1) | 152 | (17.5) | 2,095 | (73.0) | 8 | (7.5) | 54 | (4.4) | 1,512 | | (46.9) | |
| **Comorbidities*, *n (%)*** |  |  |  |  |  |  |  |  |  |  |  | |  | |
| Gynecology | 2,076 | (100.0) | 9,934 | (100.0) | 34,329 | (100.0) | 77 | (9.0) | 1,165 | (9.8) | 2,509 | | (7.2) | |
| Heavy menstrual bleeding^†^ | 692 | (33.3) | 2,762 | (27.8) | 8,599 | (25.0) | 7 | (9.1) | 66 | (5.7) | 177 | | (7.1) | |
| Abnormal uterine bleeding^†^ | 308 | (14.8) | 1,796 | (18.1) | 4,829 | (14.1) | 14 | (18.2) | 282 | (24.2) | 675 | | (26.9) | |
| Fibroids^†^ | 1,473 | (71.0) | 6,950 | (70.0) | 24,708 | (72.0) | 42 | (54.5) | 668 | (57.3) | 1,362 | | (54.3) | |
| Adenomyosis or Endometriosis^†^ | 623 | (30.0) | 2,600 | (26.2) | 8,064 | (23.5) | 15 | (19.5) | 225 | (19.3) | 452 | | (18.0) | |
| Uterine fibroid-related surgery^†^ | 185 | (8.9) | 2748 | (27.7) | 2,523 | (7.3) | 15 | (19.5) | 161 | (13.8) | 164 | | (6.5) | |
| Obstetrics | 77 | (3.7) | 1,165 | (11.7) | 2,509 | (7.3) | 858 | (100.0) | 11,929 | (100.0) | 35,069 | | (100.0) | |
| Pregnancy^†^ | 34 | (44.2) | 463 | (39.7) | 1,471 | (58.6) | 277 | (32.3) | 3,770 | (31.6) | 18,394 | | (52.5) | |
| Childbirth and the puerperium^†^ | 64 | (83.1) | 1,028 | (88.2) | 1,381 | (55.0) | 743 | (86.6) | 10,756 | (90.2) | 20,144 | | (57.4) | |
| IBD | 17 | (0.8) | 65 | (0.7) | 122 | (0.4) | 6 | (0.7) | 60 | (0.5) | 109 | | (0.3) | |
| UC^†^ | 14 | (82.4) | 48 | (73.8) | 100 | (82.0) | 3 | (50.0) | 46 | (76.7) | 93 | | (85.3) | |
| CD^†^ | 5 | (29.4) | 18 | (27.7) | 25 | (20.5) | 3 | (50.0) | 14 | (23.3) | 19 | | (17.4) | |
| Gastrointestinal bleeding  (excluding IBD) | 10 | (0.5) | 150 | (1.5) | 260 | (0.8) | 2 | (0.2) | 33 | (0.3) | 85 | | (0.2) | |
| Gastrointestinal malabsorption  (excluding IBD) | 1 | (0.0) | 22 | (0.2) | 24 | (0.1) | 0 | (0.0) | 1 | (0.0) | 0 | | (0.0) | |
| Heart failure | 31 | (1.5) | 272 | (2.7) | 582 | (1.7) | 7 | (0.8) | 70 | (0.6) | 148 | | (0.4) | |
| Cancer | 156 | (7.5) | 1,330 | (13.4) | 2,374 | (6.9) | 5 | (0.6) | 46 | (0.4) | 136 | | (0.4) | |
| NDD-CKD | 17 | (0.8) | 154 | (1.6) | 400 | (1.2) | 4 | (0.5) | 60 | (0.5) | 148 | | (0.4) | |
| **Total iron dose in the Treatment Period (mg)** | | | | | | | | | | | |  | |  |
| Median (IQR) | 500.00  (500.00–1000.00) | | 240.00  (120.00–480.00) | | 3,000.00  (1,400.00–5,600.00) | | 500.00  (500.00–500.00) | | 240.00  (160.00–480.00) | | 1,600.00  (1,200.00–2,940.00) | | |  |
| **Total IV iron dose in the Treatment Period by prescription dose (mg)** | | | | | | | | | | | | | |  |
| ≤ 500 mg*, n (%)* | 1,136 | (54.7) | 8,097 | (81.5) | - | - | 684 | (79.7) | 9,661 | (81.0) | - | | - | |
| > 500 and ≤ 1,000 mg*, n (%)* | 502 | (24.2) | 1,472 | (14.8) | - | - | 123 | (14.3) | 1,926 | (16.1) | - | | - | |
| > 1,000 and ≤ 1,500 mg*, n (%)* | 414 | (19.9) | 248 | (2.5) | - | - | 47 | (5.5) | 212 | (1.8) | - | | - | |
| > 1,500 mg*, n (%)* | 24 | (1.2) | 117 | (1.2) | - | - | 4 | (0.5) | 130 | (1.1) | - | | - | |
| **Duration in the Treatment Period (days)** | | | | | | | | | | | |  | |  |
| Median (IQR) | 1.0 (1.0–15.0) | | 3.0 (1.0–7.0) | | 30.0 (14.0–57.0) | | 1.0 (1.0–1.0) | | 3.0 (2.0–5.0) | | 15.0 (10.0–28.0) | | |  |
| **Mean iron dose per a day in the Treatment Period (mg)** | | | | | | | | | | | |  | |  |
| Median (IQR) | 500.00 (100.00–500.00) | | 80.00 (80.00–85.71) | | 100.00 (94.10–105.00) | | 500.00 (500.00–500.00) | | 80.00 (80.00–120.00) | | 100.00 (100.00–105.00) | | |  |
| **Combined with oral iron in IV iron groups from 42 days before the Index date to ETP** | | | | | | | | | | | |  | |  |
| *n (%)* | 583 | (28.1) | 4,987 | (50.2) | - | | 382 | (44.5) | 6,695 | (56.1) | - | | | |

**Table S8b. Patient characteristics in each disease area (IBD, Gastrointestinal bleeding or malabsorption [excluding IBD]).**

|  | IBD | | | | | | Gastrointestinal bleeding or malabsorption (excluding IBD) | | | | | |
| --- | --- | --- | --- | --- | --- | --- | --- | --- | --- | --- | --- | --- |
|  | FCM | | SFO | | Oral iron | | FCM | | SFO | | Oral iron | |
|  | (*N* = 607) | | (*N* = 2,077) | | (*N* = 4,249) | | (*N* = 917) | | (*N* = 24,098) | | (*N* = 21,481) | |
| **Female gender*, n (%)*** | 334 | (55.0) | 1,083 | (52.1) | 2,412 | (56.8) | 405 | (44.2) | 9,519 | (39.5) | 9,343 | (43.5) |
| **Age (years)** |  |  |  |  |  |  |  |  |  |  |  |  |
| Median (IQR) | 41.0 (29.0–52.0) | | 44.0 (29.0–59.0) | | 44.0 (32.0–61.0) | | 73.0 (62.0–83.0) | | 77.0 (67.0–84.0) | | 76.0 (66.0–84.0) | |
| < 20 years, *n (%)* | 45 | (7.4) | 220 | (10.6) | 353 | (8.3) | 7 | (0.8) | 131 | (0.5) | 368 | (1.7) |
| ≥ 20 and < 45 years, *n (%)* | 302 | (49.8) | 850 | (40.9) | 1,772 | (41.7) | 89 | (9.7) | 900 | (3.7) | 1,091 | (5.1) |
| ≥ 45 and < 55 years, *n (%)* | 132 | (21.7) | 363 | (17.5) | 790 | (18.6) | 69 | (7.5) | 1,432 | (5.9) | 1,315 | (6.1) |
| ≥ 55 and < 65 years, *n (%)* | 51 | (8.4) | 242 | (11.7) | 387 | (9.1) | 103 | (11.2) | 2,579 | (10.7) | 2,129 | (9.9) |
| ≥ 65 years, *n (%)* | 77 | (12.7) | 402 | (19.4) | 947 | (22.3) | 649 | (70.8) | 19,056 | (79.1) | 16,578 | (77.2) |
| **Weight (kg)** |  |  |  |  |  |  |  |  |  |  |  |  |
| Median (IQR) | 53.75 (46.70–61.10) | | 53.00 (45.00–62.00) | | 53.00 (45.60–62.00) | | 55.10 (46.90–65.20) | | 55.20 (47.00–64.80) | | 54.90 (46.10–64.20) | |
| *n (%)* | 136 | (22.4) | 1,446 | (69.6) | 931 | (21.9) | 643 | (70.1) | 21,690 | (90.0) | 13,824 | (64.4) |
| < 25 kg | 0 | (0.0) | 13 | (0.9) | 12 | (1.3) | 1 | (0.2) | 43 | (0.2) | 138 | (1.0) |
| ≥ 25 and < 35 kg | 2 | (1.5) | 39 | (2.7) | 33 | (3.5) | 17 | (2.6) | 626 | (2.9) | 399 | (2.9) |
| ≥ 35 and < 70 kg | 120 | (88.2) | 1,229 | (85.0) | 784 | (84.2) | 536 | (83.4) | 17,660 | (81.4) | 11,265 | (81.5) |
| ≥ 70 kg | 14 | (10.3) | 165 | (11.4) | 102 | (11.0) | 89 | (13.8) | 3,361 | (15.5) | 2,022 | (14.6) |
| **Blood transfusion within 42 days before the Index date** | | | | | | | | | | | |  |
| *n (%)* | 16 | (2.6) | 205 | (9.9) | 110 | (2.6) | 290 | (31.6) | 8,389 | (34.8) | 6,347 | (29.5) |
| > 400 mL | 10 | (62.5) | 133 | (64.9) | 67 | (60.9) | 210 | (72.4) | 5,926 | (70.6) | 4,572 | (72.0) |
| **Blood transfusion in the Treatment Period** | | | | | | | | | | | |  |
| *n (%)* | 13 | (2.1) | 288 | (13.9) | 135 | (3.2) | 147 | (16.0) | 8,417 | (34.9) | 3,441 | (16.0) |
| > 400 mL | 6 | (46.2) | 189 | (65.6) | 81 | (60.0) | 86 | (58.5) | 5,493 | (65.3) | 2,186 | (63.5) |
| **Autologous blood donation in the Treatment Period** | | | | | | | | | | | |  |
| *n (%)* | 0 | (0.0) | 8 | (0.4) | 11 | (0.3) | 2 | (0.2) | 14 | (0.1) | 48 | (0.2) |
| > 400 mL | - | | 3 | (37.5) | 4 | (36.4) | 1 | (50.0) | 8 | (57.1) | 20 | (41.7) |
| **Hb (g/dL)** |  |  |  |  |  |  |  |  |  |  |  |  |
| Median (IQR) | 9.45 (8.30–9.95) | | 8.80 (7.80–10.00) | | 9.80 (8.90–10.90) | | 8.10 (7.00–8.90) | | 8.50 (7.50–9.70) | | 8.80 (7.90–10.00) | |
| *n (%)* | 16 | (2.6) | 153 | (7.4) | 329 | (7.7) | 192 | (20.9) | 2,817 | (11.7) | 2,506 | (11.7) |
| < 8 g/dL | 2 | (12.5) | 47 | (30.7) | 37 | (11.2) | 91 | (47.4) | 1,030 | (36.6) | 685 | (27.3) |
| ≥ 8 g/dL | 14 | (87.5) | 106 | (69.3) | 292 | (88.8) | 101 | (52.6) | 1,787 | (63.4) | 1,821 | (72.7) |
| < 10 g/dL | 12 | (75.0) | 112 | (73.2) | 178 | (54.1) | 175 | (91.1) | 2,237 | (79.4) | 1,873 | (74.7) |
| ≥ 10 g/dL | 4 | (25.0) | 41 | (26.8) | 151 | (45.9) | 17 | (8.9) | 580 | (20.6) | 633 | (25.3) |
| **Serum ferritin (ng/mL)** |  |  |  |  |  |  |  |  |  |  |  |  |
| Median (IQR) | 14.00 (5.00–94.00) | | 35.40 (8.00–100.70) | | 17.85 (6.10–37.65) | | 32.80 (14.00–99.00) | | 33.00 (13.50–100.10) | | 31.95 (12.30–88.00) | |
| *n (%)* | 7 | (1.2) | 51 | (2.5) | 100 | (2.4) | 57 | (6.2) | 838 | (3.5) | 818 | (3.8) |
| < 12 ng/mL | 3 | (42.9) | 13 | (25.5) | 47 | (47.0) | 10 | (17.5) | 178 | (21.2) | 193 | (23.6) |
| ≥ 12 ng/mL | 4 | (57.1) | 38 | (74.5) | 53 | (53.0) | 47 | (82.5) | 660 | (78.8) | 625 | (76.4) |
| **Serum iron (µg/dL)** |  |  |  |  |  |  |  |  |  |  |  |  |
| Median (IQR) | 16.00 (11.00–19.00) | | 17.00 (10.00–29.00) | | 22.00 (13.00–32.00) | | 30.00 (15.00–73.00) | | 32.00 (16.00–69.00) | | 29.00 (17.00–53.00) | |
| *n (%)* | 11 | (1.8) | 89 | (4.3) | 159 | (3.7) | 91 | (9.9) | 1,441 | (6.0) | 1,265 | (5.9) |
| **TSAT (%)** |  |  |  |  |  |  |  |  |  |  |  |  |
| Median (IQR) | 5.77 (2.80–8.49) | | 4.84 (3.23–11.98) | | 6.13 (3.81–11.19) | | 10.80 (5.57–32.50) | | 13.01 (6.11–27.41) | | 9.81 (5.32–21.31) | |
| *n (%)* | 10 | (1.6) | 53 | (2.6) | 113 | (2.7) | 75 | (8.2) | 950 | (3.9) | 868 | (4.0) |
| < 20% | 10 | (100.0) | 46 | (86.8) | 98 | (86.7) | 50 | (66.7) | 611 | (64.3) | 637 | (73.4) |
| ≥ 20% | 0 | (0.0) | 7 | (13.2) | 15 | (13.3) | 25 | (33.3) | 339 | (35.7) | 231 | (26.6) |
| **CRP (mg/dL)** |  |  |  |  |  |  |  |  |  |  |  |  |
| *n (%)* | 14 | (2.3) | 143 | (6.9) | 272 | (6.4) | 182 | (19.8) | 2,688 | (11.2) | 2,198 | (10.2) |
| ≤ 1.0 mg/dL | 11 | (78.6) | 58 | (40.6) | 183 | (67.3) | 124 | (68.1) | 1,700 | (63.2) | 1,513 | (68.8) |
| > 1.0 mg/dL | 3 | (21.4) | 85 | (59.4) | 89 | (32.7) | 58 | (31.9) | 988 | (36.8) | 685 | (31.2) |
| **Inpatient or Outpatient*, n (%)*** |  |  |  |  |  |  |  |  |  |  |  |  |
| Inpatient | 104 | (17.1) | 1,464 | (70.5) | 471 | (11.1) | 580 | (63.2) | 23,019 | (95.5) | 7,668 | (35.7) |
| Outpatient | 503 | (82.9) | 613 | (29.5) | 3,778 | (88.9) | 337 | (36.8) | 1,079 | (4.5) | 13,813 | (64.3) |
| **"Inpatient or Outpatient" and "Hb ≥ 8 or < 8 g/dL", n (%)^‡^** | | | | | | | | | | | |  |
| Inpatient and Hb < 8 g/dL | 1 | (6.3) | 36 | (23.5) | 8 | (2.4) | 71 | (37.0) | 989 | (35.1) | 304 | (12.1) |
| Inpatient and Hb ≥ 8 g/dL | 6 | (37.5) | 85 | (55.6) | 40 | (12.2) | 89 | (46.4) | 1,710 | (60.7) | 656 | (26.2) |
| Outpatient and Hb < 8 g/dL | 1 | (6.3) | 11 | (7.2) | 29 | (8.8) | 20 | (10.4) | 41 | (1.5) | 381 | (15.2) |
| Outpatient and Hb ≥8 g/dL | 8 | (50.0) | 21 | (13.7) | 252 | (76.6) | 12 | (6.3) | 77 | (2.7) | 1,165 | (46.5) |
| **Comorbidities*, *n (%)*** |  |  |  |  |  |  |  |  |  |  |  |  |
| Gynecology | 17 | (2.8) | 65 | (3.1) | 122 | (2.9) | 11 | (1.2) | 168 | (0.7) | 282 | (1.3) |
| Heavy menstrual bleeding^†^ | 3 | (17.6) | 14 | (21.5) | 30 | (24.6) | 4 | (36.4) | 23 | (13.7) | 51 | (18.1) |
| Abnormal uterine bleeding^†^ | 1 | (5.9) | 13 | (20.0) | 14 | (11.5) | 2 | (18.2) | 37 | (22.0) | 39 | (13.8) |
| Fibroids^†^ | 8 | (47.1) | 41 | (63.1) | 78 | (63.9) | 8 | (72.7) | 118 | (70.2) | 194 | (68.8) |
| Adenomyosis or Endometriosis^†^ | 7 | (41.2) | 23 | (35.4) | 26 | (21.3) | 2 | (18.2) | 24 | (14.3) | 61 | (21.6) |
| Uterine fibroid-related surgery^†^ | 0 | (0.0) | 16 | (24.6) | 4 | (3.3) | 0 | (0.0) | 14 | (8.3) | 12 | (4.3) |
| Obstetrics | 6 | (1.0) | 60 | (2.9) | 109 | (2.6) | 2 | (0.2) | 34 | (0.1) | 85 | (0.4) |
| Pregnancy^†^ | 3 | (50.0) | 27 | (45.0) | 72 | (66.1) | 1 | (50.0) | 18 | (52.9) | 48 | (56.5) |
| Childbirth and the puerperium^†^ | 5 | (83.3) | 52 | (86.7) | 47 | (43.1) | 1 | (50.0) | 33 | (97.1) | 47 | (55.3) |
| IBD | 607 | (100.0) | 2,077 | (100.0) | 4,249 | (100.0) | 112 | (12.2) | 439 | (1.8) | 513 | (2.4) |
| UC^†^ | 319 | (52.6) | 1,433 | (69.0) | 3,163 | (74.4) | 62 | (55.4) | 295 | (67.2) | 384 | (74.9) |
| CD^†^ | 319 | (52.6) | 706 | (34.0) | 1,214 | (28.6) | 56 | (50.0) | 157 | (35.8) | 144 | (28.1) |
| Gastrointestinal or | 95 | (15.7) | 382 | (18.4) | 447 | (10.5) | 852 | (92.9) | 22,862 | (94.9) | 20,143 | (93.8) |
| malabsorption (excluding IBD) | 21 | (3.5) | 67 | (3.2) | 75 | (1.8) | 75 | (8.2) | 1,481 | (6.1) | 1,491 | (6.9) |
| Heart failure | 31 | (5.1) | 132 | (6.4) | 286 | (6.7) | 172 | (18.8) | 4,412 | (18.3) | 4,649 | (21.6) |
| Cancer | 18 | (3.0) | 193 | (9.3) | 312 | (7.3) | 263 | (28.7) | 6,538 | (27.1) | 6,571 | (30.6) |
| NDD-CKD | 19 | (3.1) | 93 | (4.5) | 194 | (4.6) | 75 | (8.2) | 2,225 | (9.2) | 2,324 | (10.8) |
| **Total iron dose in the Treatment Period (mg)** | | | | | | | | | | | |  |
| Median (IQR) | 500.00  (500.00–1,000.00) | | 360.00  (160.00–680.00) | | 5,250.00  (2,800.00–9,800.00) | | 500.00  (500.00–1,000.00) | | 320.00  (200.00–560.00) | | 3,150.00  (1,470.00–6,615.00) | |
| **Total IV iron dose in the Treatment Period by prescription dose (mg)** | | | | | | | | | | | | |
| ≤ 500 mg*, n (%)* | 376 | (61.9) | 1313 | (63.2) | - | - | 582 | (63.5) | 17492 | (72.6) | - | - |
| > 500 and ≤ 1,000 mg*, n (%)* | 121 | (19.9) | 485 | (23.4) | - | - | 163 | (17.8) | 4855 | (20.1) | - | - |
| > 1,000 and ≤ 1,500 mg*, n (%)* | 94 | (15.5) | 165 | (7.9) | - | - | 156 | (17.0) | 1115 | (4.6) | - | - |
| > 1,500 mg*, n (%)* | 16 | (2.6) | 114 | (5.5) | - | - | 16 | (1.7) | 636 | (2.6) | - | - |
| **Duration in the Treatment Period (days)** | | | | | | | | | | | |  |
| Median (IQR) | 1.0 (1.0–15.0) | | 7.0 (3.0–14.0) | | 56.0 (28.0–112.0) | | 1.0 (1.0–8.0) | | 5.0 (3.0–8.0) | | 31.0 (15.0–64.0) | |
| **Mean iron dose per a day in the Treatment Period (mg)** | | | | | | | | | | | |  |
| Median (IQR) | 500.00 (100.00–500.00) | | 80.00 (40.00–80.00) | | 100.00 (53.85–104.56) | | 500.00 (125.00–500.00) | | 80.00 (40.00–80.00) | | 100.00 (92.46–111.36) | |
| **Combined with oral iron in IV iron groups from 42 days before the Index date to ETP** | | | | | | | | | | | |  |
| *n (%)* | 192 | (31.6) | 596 | (28.7) | - | | 190 | (20.7) | 5,079 | (21.1) | - | |

**Table S8c. Patient characteristics in each disease area (Heart failure, Cancer).**

|  | Heart failure | | | | | | Cancer | | | | | |
| --- | --- | --- | --- | --- | --- | --- | --- | --- | --- | --- | --- | --- |
|  | FCM | | SFO | | Oral iron | | FCM | | SFO | | Oral iron | |
|  | (*N* = 803) | | (*N* = 18,044) | | (*N* = 60,666) | | (*N* = 1,503) | | (*N* = 26,258) | | (*N* = 64,805) | |
| **Female gender*, n (%)*** | 452 | (56.3) | 8,916 | (49.4) | 32,042 | (52.8) | 859 | (57.2) | 12,220 | (46.5) | 31,932 | (49.3) |
| **Age (years)** |  |  |  |  |  |  |  |  |  |  |  |  |
| Median (IQR) | 80.0 (71.0–87.0) | | 81.0 (73.0–87.0) | | 82.0 (73.0–88.0) | | 71.0 (58.0–79.0) | | 75.0 (67.0–82.0) | | 75.0 (66.0–82.0) | |
| < 20 years, *n (%)* | 1 | (0.1) | 48 | (0.3) | 709 | (1.2) | 1 | (0.1) | 17 | (0.1) | 70 | (0.1) |
| ≥ 20 and < 45 years, *n (%)* | 32 | (4.0) | 418 | (2.3) | 1,490 | (2.5) | 89 | (5.9) | 915 | (3.5) | 2,791 | (4.3) |
| ≥ 45 and < 55 years, *n (%)* | 45 | (5.6) | 620 | (3.4) | 2,183 | (3.6) | 222 | (14.8) | 1,925 | (7.3) | 5,146 | (7.9) |
| ≥ 55 and < 65 years, *n (%)* | 41 | (5.1) | 1,057 | (5.9) | 2,908 | (4.8) | 204 | (13.6) | 2,803 | (10.7) | 6,186 | (9.5) |
| ≥ 65 years, *n (%)* | 684 | (85.2) | 15,901 | (88.1) | 53,376 | (88.0) | 987 | (65.7) | 20,598 | (78.4) | 50,612 | (78.1) |
| **Weight (kg)** |  |  |  |  |  |  |  |  |  |  |  |  |
| Median (IQR) | 51.00 (44.20–60.70) | | 53.00 (44.40–62.00) | | 51.00 (43.20–60.30) | | 53.60 (46.50–62.00) | | 53.30 (45.80–61.80) | | 53.30 (46.00–61.80) | |
| *n (%)* | 451 | (56.2) | 15,120 | (83.8) | 34,719 | (57.2) | 830 | (55.2) | 22,712 | (86.5) | 34,184 | (52.7) |
| < 25 kg | 0 | (0.0) | 41 | (0.3) | 313 | (0.9) | 0 | (0.0) | 8 | (0.0) | 23 | (0.1) |
| ≥ 25 and < 35 kg | 18 | (4.0) | 692 | (4.6) | 1,721 | (5.0) | 21 | (2.5) | 698 | (3.1) | 926 | (2.7) |
| ≥ 35 and < 70 kg | 395 | (87.6) | 12,547 | (83.0) | 29,353 | (84.5) | 722 | (87.0) | 19,577 | (86.2) | 29,666 | (86.8) |
| ≥ 70 kg | 38 | (8.4) | 1,840 | (12.2) | 3,332 | (9.6) | 87 | (10.5) | 2,429 | (10.7) | 3,569 | (10.4) |
| **Blood transfusion within 42 days before the Index date** | | | | | | | | | | | |  |
| *n (%)* | 170 | (21.2) | 3,900 | (21.6) | 6,069 | (10.0) | 285 | (19.0) | 6,641 | (25.3) | 6,793 | (10.5) |
| > 400 mL | 99 | (58.2) | 2536 | (65.0) | 3,716 | (61.2) | 200 | (70.2) | 4,573 | (68.9) | 4,422 | (65.1) |
| **Blood transfusion in the Treatment Period** | | | | | | | | | | | |  |
| *n (%)* | 99 | (12.3) | 5,022 | (27.8) | 7,668 | (12.6) | 148 | (9.8) | 6,957 | (26.5) | 9,966 | (15.4) |
| > 400 mL | 44 | (44.4) | 3,127 | (62.3) | 4,449 | (58.0) | 86 | (58.1) | 4,438 | (63.8) | 6,521 | (65.4) |
| **Autologous blood donation in the Treatment Period** | | | | | | |  | | | | | |
| *n (%)* | 15 | (1.9) | 190 | (1.1) | 546 | (0.9) | 74 | (4.9) | 325 | (1.2) | 1,548 | (2.4) |
| > 400 mL | 3 | (20.0) | 97 | (51.1) | 284 | (52.0) | 20 | (27.0) | 174 | (53.5) | 679 | (43.9) |
| **Hb (g/dL)** |  |  |  |  |  |  |  |  |  |  |  |  |
| Median (IQR) | 8.40 (7.40–9.30) | | 8.90 (7.70–10.30) | | 9.40 (8.30–10.70) | | 8.30 (7.20–9.20) | | 8.50 (7.50–9.60) | | 9.30 (8.20–10.70) | |
| *n (%)* | 117 | (14.6) | 2,474 | (13.7) | 6,876 | (11.3) | 195 | (13.0) | 3,164 | (12.0) | 7,627 | (11.8) |
| < 8 g/dL | 47 | (40.2) | 761 | (30.8) | 1,214 | (17.7) | 80 | (41.0) | 1,133 | (35.8) | 1,563 | (20.5) |
| ≥ 8 g/dL | 70 | (59.8) | 1,713 | (69.2) | 5,662 | (82.3) | 115 | (59.0) | 2,031 | (64.2) | 6,064 | (79.5) |
| < 10 g/dL | 98 | (83.8) | 1,715 | (69.3) | 4,308 | (62.7) | 173 | (88.7) | 2,567 | (81.1) | 4,856 | (63.7) |
| ≥ 10 g/dL | 19 | (16.2) | 759 | (30.7) | 2,568 | (37.3) | 22 | (11.3) | 597 | (18.9) | 2,771 | (36.3) |
| **Serum ferritin (ng/mL)** |  | | | | | |  | | | | | |
| Median (IQR) | 25.20 (14.00–76.00) | | 34.60 (14.70–101.70) | | 30.10 (14.00–84.70) | | 18.50 (8.00–87.00) | | 29.95 (11.00–96.95) | | 26.10 (10.50–99.10) | |
| *n (%)* | 54 | (6.7) | 805 | (4.5) | 2,448 | (4.0) | 56 | (3.7) | 872 | (3.3) | 1,913 | (3.0) |
| < 12 ng/mL | 12 | (22.2) | 152 | (18.9) | 483 | (19.7) | 20 | (35.7) | 235 | (26.9) | 541 | (28.3) |
| ≥ 12 ng/mL | 42 | (77.8) | 653 | (81.1) | 1,965 | (80.3) | 36 | (64.3) | 637 | (73.1) | 1,372 | (71.7) |
| **Serum iron (µg/dL)** |  | | | | | |  | | | | | |
| Median (IQR) | 22.00 (13.00–39.00) | | 25.00 (15.00–44.00) | | 28.00 (19.00–45.00) | | 22.00 (11.00–48.00) | | 22.00 (13.00–41.00) | | 27.00 (16.00–45.00) | |
| *n (%)* | 77 | (9.6) | 1,320 | (7.3) | 3,708 | (6.1) | 83 | (5.5) | 1,630 | (6.2) | 3,071 | (4.7) |
| **TSAT (%)** |  |  |  |  |  |  |  |  |  |  |  |  |
| Median (IQR) | 7.36 (4.44–11.89) | | 9.12 (4.79–18.68) | | 9.31 (5.60–16.49) | | 6.21 (3.68–13.92) | | 8.43 (4.50–18.18) | | 8.58 (4.73–16.44) | |
| *n (%)* | 65 | (8.1) | 803 | (4.5) | 2,497 | (4.1) | 59 | (3.9) | 1,039 | (4.0) | 2,056 | (3.2) |
| < 20% | 54 | (83.1) | 620 | (77.2) | 2,045 | (81.9) | 49 | (83.1) | 810 | (78.0) | 1,664 | (80.9) |
| ≥ 20% | 11 | (16.9) | 183 | (22.8) | 452 | (18.1) | 10 | (16.9) | 229 | (22.0) | 392 | (19.1) |
| **CRP (mg/dL)** |  |  |  |  |  |  |  |  |  |  |  |  |
| *n (%)* | 108 | (13.4) | 2,183 | (12.1) | 5,583 | (9.2) | 176 | (11.7) | 2,914 | (11.1) | 6,236 | (9.6) |
| ≤ 1.0 mg/dL | 70 | (64.8) | 1,266 | (58.0) | 3,376 | (60.5) | 89 | (50.6) | 1,044 | (35.8) | 3,648 | (58.5) |
| > 1.0 mg/dL | 38 | (35.2) | 917 | (42.0) | 2,207 | (39.5) | 87 | (49.4) | 1,870 | (64.2) | 2,588 | (41.5) |
| **Inpatient or Outpatient*, n (%)*** |  | | | | | |  | | | | | |
| Inpatient | 433 | (53.9) | 15,881 | (88.0) | 25,375 | (41.8) | 725 | (48.2) | 23,285 | (88.7) | 20,220 | (31.2) |
| Outpatient | 370 | (46.1) | 2,163 | (12.0) | 35,291 | (58.2) | 778 | (51.8) | 2,973 | (11.3) | 44,585 | (68.8) |
| **"Inpatient or Outpatient" and "Hb ≥ 8 or < 8 g/dL", n (%)^‡^** | | | | | | |  | | | | | |
| Inpatient and Hb < 8 g/dL | 25 | (21.4) | 676 | (27.3) | 540 | (7.9) | 51 | (26.2) | 1,018 | (32.2) | 488 | (6.4) |
| Inpatient and Hb ≥ 8 g/dL | 40 | (34.2) | 1,579 | (63.8) | 2,428 | (35.3) | 79 | (40.5) | 1,851 | (58.5) | 1,970 | (25.8) |
| Outpatient and Hb < 8 g/dL | 22 | (18.8) | 85 | (3.4) | 674 | (9.8) | 29 | (14.9) | 115 | (3.6) | 1,075 | (14.1) |
| Outpatient and Hb ≥ 8 g/dL | 30 | (25.6) | 134 | (5.4) | 3,234 | (47.0) | 36 | (18.5) | 180 | (5.7) | 4,094 | (53.7) |
| **Comorbidities*, *n (%)*** |  | | | | | |  | | | | | |
| Gynecology | 31 | (3.9) | 272 | (1.5) | 582 | (1.0) | 156 | (10.4) | 1,330 | (5.1) | 2374 | (3.7) |
| Heavy menstrual bleeding^†^ | 13 | (41.9) | 73 | (26.8) | 134 | (23.0) | 26 | (16.7) | 183 | (13.8) | 324 | (13.6) |
| Abnormal uterine bleeding^†^ | 10 | (32.3) | 52 | (19.1) | 116 | (19.9) | 65 | (41.7) | 488 | (36.7) | 762 | (32.1) |
| Fibroids^†^ | 20 | (64.5) | 194 | (71.3) | 384 | (66.0) | 85 | (54.5) | 701 | (52.7) | 1,367 | (57.6) |
| Adenomyosis or Endometriosis^†^ | 7 | (22.6) | 49 | (18.0) | 95 | (16.3) | 18 | (11.5) | 175 | (13.2) | 279 | (11.8) |
| Uterine fibroid-related surgery^†^ | 0 | (0.0) | 30 | (11.0) | 32 | (5.5) | 10 | (6.4) | 192 | (14.4) | 182 | (7.7) |
| Obstetrics | 7 | (0.9) | 70 | (0.4) | 148 | (0.2) | 5 | (0.3) | 46 | (0.2) | 136 | (0.2) |
| Pregnancy^†^ | 4 | (57.1) | 29 | (41.4) | 77 | (52.0) | 4 | (80.0) | 26 | (56.5) | 84 | (61.8) |
| Childbirth and the puerperium^†^ | 4 | (57.1) | 61 | (87.1) | 89 | (60.1) | 2 | (40.0) | 38 | (82.6) | 71 | (52.2) |
| IBD | 31 | (3.9) | 132 | (0.7) | 286 | (0.5) | 18 | (1.2) | 193 | (0.7) | 312 | (0.5) |
| UC^†^ | 20 | (64.5) | 103 | (78.0) | 240 | (83.9) | 12 | (66.7) | 165 | (85.5) | 253 | (81.1) |
| CD^†^ | 13 | (41.9) | 31 | (23.5) | 60 | (21.0) | 9 | (50.0) | 36 | (18.7) | 66 | (21.2) |
| Gastrointestinal bleeding  (excluding IBD) | 159 | (19.8) | 4,245 | (23.5) | 4435 | (7.3) | 228 | (15.2) | 5,661 | (21.6) | 5,775 | (8.9) |
| Gastrointestinal malabsorption  (excluding IBD) | 13 | (1.6) | 207 | (1.1) | 245 | (0.4) | 39 | (2.6) | 1,046 | (4.0) | 896 | (1.4) |
| Heart failure | 803 | (100.0) | 18,044 | (100.0) | 60,666 | (100.0) | 188 | (12.5) | 4,105 | (15.6) | 10,821 | (16.7) |
| Cancer | 188 | (23.4) | 4,105 | (22.7) | 10,821 | (17.8) | 1,503 | (100.0) | 26,258 | (100.0) | 64,805 | (100.0) |
| NDD-CKD | 222 | (27.6) | 5,158 | (28.6) | 17,571 | (29.0) | 76 | (5.1) | 2,364 | (9.0) | 6,489 | (10.0) |
| **Total iron dose in the Treatment Period (mg)** | | | | | | |  | | | | | |
| Median (IQR) | 500.00  (500.00–1,000.00) | | 240.00  (120.00–480.00) | | 3,500.00  (1,440.00–8,400.00) | | 500.00  (500.00–1,000.00) | | 320.00  (160.00–600.00) | | 3,300.00  (1,400.00–8,000.00) | |
| **Total IV iron dose in the Treatment Period by prescription dose (mg)** | | | | | | | | | | | | |
| ≤ 500 mg*, n (%)* | 533 | (66.4) | 13,764 | (76.3) | - | - | 932 | (62.0) | 17,642 | (67.2) | - | - |
| > 500 and ≤ 1,000 mg*, n (%)* | 146 | (18.2) | 3,127 | (17.3) | - | - | 320 | (21.3) | 5,735 | (21.8) | - | - |
| > 1,000 and ≤ 1,500 mg*, n (%)* | 110 | (13.7) | 702 | (3.9) | - | - | 222 | (14.8) | 1,626 | (6.2) | - | - |
| > 1500 mg*, n (%)* | 14 | (1.7) | 451 | (2.5) | - | - | 29 | (1.9) | 1,255 | (4.8) | - | - |
| **Duration in the Treatment Period (days)** | | | | | | |  | | | | | |
| Median (IQR) | 1.0 (1.0–8.0) | | 5.0 (3.0–9.0) | | 38.0 (17.0–91.0) | | 1.0 (1.0–9.0) | | 6.0 (3.0–10.0) | | 32.0 (14.0–78.0) | |
| **Mean iron dose per a day in the Treatment Period (mg)** | | | | | | |  | | | | | |
| Median (IQR) | 500.00 (125.00–500.00) | | 60.00 (40.00–80.00) | | 100.00 (52.50–105.00) | | 500.00 (111.11–500.00) | | 80.00 (40.00–80.00) | | 100.00 (86.80–113.75) | |
| **Combined with oral iron in IV iron groups from 42 days before the Index date to ETP** | | | | | | |  | | | | | |
| *n (%)* | 216 | (26.9) | 5,063 | (28.1) | - | | 395 | (26.3) | 7,826 | (29.8) | - | |

**Table S8d. Patient characteristics in each disease area (NDD-CKD).**

|  | NDD-CKD | | | | | |
| --- | --- | --- | --- | --- | --- | --- |
|  | FCM | | SFO | | Oral iron | |
|  | (*N* = 381) | | (*N* = 10,867) | | (*N* = 37,994) | |
| **Female gender*, n (%)*** | 213 | (55.9) | 4,736 | (43.6) | 18,278 | (48.1) |
| **Age (years)** |  |  |  |  |  |  |
| Median (IQR) | 78.0 (69.0–86.0) | | 79.0 (70.0–86.0) | | 79.0 (70.0–86.0) | |
| < 20 years, *n (%)* | 0 | (0.0) | 8 | (0.1) | 137 | (0.4) |
| ≥ 20 and < 45 years, *n (%)* | 18 | (4.7) | 314 | (2.9) | 1,508 | (4.0) |
| ≥ 45 and < 55 years, *n (%)* | 34 | (8.9) | 496 | (4.6) | 1,961 | (5.2) |
| ≥ 55 and < 65 years, *n (%)* | 26 | (6.8) | 897 | (8.3) | 2,642 | (7.0) |
| ≥ 65 years, *n (%)* | 303 | (79.5) | 9,152 | (84.2) | 31,746 | (83.6) |
| **Weight (kg)** |  |  |  |  |  |  |
| Median (IQR) | 53.70 (46.40–64.15) | | 54.50 (46.10–63.10) | | 54.00 (46.00–63.00) | |
| *n (%)* | 192 | (50.4) | 8,729 | (80.3) | 20,209 | (53.2) |
| < 25 kg | 0 | (0.0) | 8 | (0.1) | 24 | (0.1) |
| ≥ 25 and < 35 kg | 4 | (2.1) | 285 | (3.3) | 604 | (3.0) |
| ≥ 35 and < 70 kg | 160 | (83.3) | 7,256 | (83.1) | 16,942 | (83.8) |
| ≥ 70 kg | 28 | (14.6) | 1,180 | (13.5) | 2,639 | (13.1) |
| **Blood transfusion within 42 days before the Index date** | | | | | |  |
| *n (%)* | 64 | (16.8) | 1,919 | (17.7) | 2,659 | (7.0) |
| > 400 mL | 44 | (68.8) | 1,202 | (62.6) | 1,552 | (58.4) |
| **Blood transfusion in the Treatment Period** | | | | | |  |
| *n (%)* | 39 | (10.2) | 2,552 | (23.5) | 4,406 | (11.6) |
| > 400 mL | 17 | (43.6) | 1,534 | (60.1) | 2,590 | (58.8) |
| **Autologous blood donation in the Treatment Period** | | | | | |  |
| *n (%)* | 3 | (0.8) | 239 | (2.2) | 378 | (1.0) |
| > 400 mL | 2 | (66.7) | 163 | (68.2) | 211 | (55.8) |
| **Hb (g/dL)** |  |  |  |  |  |  |
| Median (IQR) | 8.10 (7.60–8.90) | | 8.60 (7.40–10.00) | | 9.50 (8.30–10.80) | |
| *n (%)* | 41 | (10.8) | 1,094 | (10.1) | 4,068 | (10.7) |
| < 8 g/dL | 18 | (43.9) | 406 | (37.1) | 730 | (17.9) |
| ≥ 8 g/dL | 23 | (56.1) | 688 | (62.9) | 3,338 | (82.1) |
| < 10 g/dL | 40 | (97.6) | 810 | (74.0) | 2,481 | (61.0) |
| ≥ 10 g/dL | 1 | (2.4) | 284 | (26.0) | 1,587 | (39.0) |
| **Serum ferritin (ng/mL)** |  | | | | | |
| Median (IQR) | 36.00 (17.25–80.95) | | 45.25 (20.80–121.40) | | 41.00 (18.70–100.00) | |
| *n (%)* | 16 | (4.2) | 426 | (3.9) | 1,531 | (4.0) |
| < 12 ng/mL | 1 | (6.3) | 46 | (10.8) | 197 | (12.9) |
| ≥ 12 ng/mL | 15 | (93.8) | 380 | (89.2) | 1,334 | (87.1) |
| **Serum iron (µg/dL)** |  | | | | |  |
| Median (IQR) | 41.00 (23.00–61.00) | | 29.00 (18.00–48.00) | | 34.00 (22.00–56.00) | |
| *n (%)* | 24 | (6.3) | 636 | (5.9) | 2,201 | (5.8) |
| **TSAT (%)** |  |  |  |  |  |  |
| Median (IQR) | 15.04 (8.36–25.17) | | 11.62 (6.38–21.35) | | 12.28 (7.02–20.43) | |
| *n (%)* | 21 | (5.5) | 422 | (3.9) | 1,547 | (4.1) |
| < 20% | 13 | (61.9) | 299 | (70.9) | 1,142 | (73.8) |
| ≥ 20% | 8 | (38.1) | 123 | (29.1) | 405 | (26.2) |
| **CRP (mg/dL)** |  |  |  |  |  |  |
| *n (%)* | 39 | (10.2) | 972 | (8.9) | 3,228 | (8.5) |
| ≤ 1.0 mg/dL | 25 | (64.1) | 515 | (53.0) | 2,002 | (62.0) |
| > 1.0 mg/dL | 14 | (35.9) | 457 | (47.0) | 1,226 | (38.0) |
| **Inpatient or Outpatient*, n (%)*** |  | | | | |  |
| Inpatient | 177 | (46.5) | 8,911 | (82.0) | 15,142 | (39.9) |
| Outpatient | 204 | (53.5) | 1,956 | (18.0) | 22,852 | (60.1) |
| **"Inpatient or Outpatient" and "Hb ≥ 8 or < 8 g/dL", n (%)^‡^** | | | | | | |
| Inpatient and Hb < 8 g/dL | 13 | (31.7) | 347 | (31.7) | 333 | (8.2) |
| Inpatient and Hb ≥ 8 g/dL | 12 | (29.3) | 595 | (54.4) | 1,354 | (33.3) |
| Outpatient and Hb < 8 g/dL | 5 | (12.2) | 59 | (5.4) | 397 | (9.8) |
| Outpatient and Hb ≥ 8 g/dL | 11 | (26.8) | 93 | (8.5) | 1,984 | (48.8) |
| **Comorbidities*, *n (%)*** |  | | | | |  |
| Gynecology | 17 | (4.5) | 154 | (1.4) | 400 | (1.1) |
| Heavy menstrual bleeding^†^ | 4 | (23.5) | 34 | (22.1) | 77 | (19.3) |
| Abnormal uterine bleeding^†^ | 2 | (11.8) | 22 | (14.3) | 71 | (17.8) |
| Fibroids^†^ | 12 | (70.6) | 96 | (62.3) | 270 | (67.5) |
| Adenomyosis or Endometriosis^†^ | 2 | (11.8) | 30 | (19.5) | 71 | (17.8) |
| Uterine fibroid-related surgery^†^ | 2 | (11.8) | 21 | (13.6) | 19 | (4.8) |
| Obstetrics | 4 | (1.0) | 60 | (0.6) | 148 | (0.4) |
| Pregnancy^†^ | 2 | (50.0) | 17 | (28.3) | 87 | (58.8) |
| Childbirth and the puerperium^†^ | 4 | (100.0) | 54 | (90.0) | 78 | (52.7) |
| IBD | 19 | (5.0) | 93 | (0.9) | 194 | (0.5) |
| UC^†^ | 11 | (57.9) | 64 | (68.8) | 145 | (74.7) |
| CD^†^ | 11 | (57.9) | 34 | (36.6) | 55 | (28.4) |
| Gastrointestinal bleeding  (excluding IBD) | 70 | (18.4) | 2,123 | (19.5) | 2224 | (5.9) |
| Gastrointestinal malabsorption  (excluding IBD) | 6 | (1.6) | 122 | (1.1) | 116 | (0.3) |
| Heart failure | 222 | (58.3) | 5,158 | (47.5) | 17,571 | (46.2) |
| Cancer | 76 | (19.9) | 2,364 | (21.8) | 6,489 | (17.1) |
| NDD-CKD | 381 | (100.0) | 10,867 | (100.0) | 37,994 | (100.0) |
| **Total iron dose in the Treatment Period (mg)** | | | | | |  |
| Median (IQR) | 500.00 (500.00–1,000.00) | | 200.00 (80.00–400.00) | | 3,600.00 (1,400.00–8,928.00) | |
| **Total IV iron dose in the Treatment Period by prescription dose (mg)** | | | | | | |
| ≤ 500 mg*, n (%)* | 250 | (65.6) | 8,828 | (81.2) | - | - |
| > 500 and ≤ 1,000 mg*, n (%)* | 63 | (16.5) | 1,466 | (13.5) | - | - |
| > 1,000 and ≤ 1,500 mg*, n (%)* | 59 | (15.5) | 346 | (3.2) | - | - |
| > 1500 mg*, n (%)* | 9 | (2.4) | 227 | (2.1) | - | - |
| **Duration in the Treatment Period (days)** | | | | | |  |
| Median (IQR) | 1.0 (1.0–8.0) | | 6.0 (2.0–12.0) | | 40.0 (16.0–100.0) | |
| **Mean iron dose per a day in the Treatment Period (mg)** | | | | | | |
| Median (IQR) | 500.00 (125.00–500.00) | | 40.00 (21.18–80.00) | | 100.00 (51.00–106.86) | |
| **Combined with oral iron in IV iron groups from 42 days before the Index date to ETP** | | | | | | |
| *n (%)* | 124 | (32.5) | 2,986 | (27.5) | - | |

Abbreviations: CD, Crohn's disease; CRP, C-reactive protein; ETP, The end date of Treatment Period; FCM, ferric carboxymaltose; Hb, hemoglobin; IBD, inflammatory bowel disease; IQR, interquartile range; IV, intravenous; NDD-CKD, non-dialysis dependent chronic kidney disease; SFO, saccharated ferric oxide; TSAT, transferrin saturation; UC, ulcerative colitis.

*Multiple choice allowed.

^†^The denominator of n (%) is the patients who applied superordinate disease area in each iron group.

^‡^The denominator of n (%) is the total number of patients in each iron group that exhibit both of the following conditions: (1) The patients are classified as Inpatient or Outpatient. (2) The patients are classified as Hb ≥ 8 g/dL or Hb < 8 g/dL.
